# Emergence of genotype Cosmopolitan of dengue virus type 2 and genotype III of dengue virus type 3 in Thailand

**DOI:** 10.1371/journal.pone.0207220

**Published:** 2018-11-12

**Authors:** Juthamas Phadungsombat, Marco Yung-Cheng Lin, Narinee Srimark, Atsushi Yamanaka, Emi E. Nakayama, Visal Moolasart, Patama Suttha, Tatsuo Shioda, Sumonmal Uttayamakul

**Affiliations:** 1 Mahidol-Osaka Center for Infectious Diseases (MOCID), Faculty of Tropical Medicine, Mahidol University, Bangkok, Thailand; 2 Department of Medical Research, Mackay Memorial Hospital, New Taipei, Taiwan; 3 Research Institute for Microbial Diseases, Osaka University, Suita, Japan; 4 Bamrasnaradura Infectious Diseases Institute, Nonthaburi, Thailand; Faculty of Science, Ain Shams University (ASU), EGYPT

## Abstract

Dengue is a mosquito-borne disease that has spread to over 100 countries. Dengue fever is caused by dengue virus (DENV), which belongs to the *Flavivirus* genus of the family *Flaviviridae*. DENV comprises 4 serotypes (DENV-1 to DENV-4), and each serotype is divided into distinct genotypes. Thailand is an endemic area where all 4 serotypes of DENV co-circulate. To understand the current genotype distribution of DENVs in Thailand, we enrolled 100 cases of fever with dengue-like symptoms at the Bamrasnaradura Infectious Diseases Institute during 2016–2017. Among them, 37 cases were shown to be dengue-positive by real-time PCR. We were able to isolate DENVs from 21 cases, including 1 DENV-1, 8 DENV-2, 4 DENV-3, and 8 DENV-4. To investigate the divergence of the viruses, RNA was extracted from isolated DENVs and viral near-whole genome sequences were determined. Phylogenetic analysis of the obtained viral sequences revealed that DENV-2 genotype Cosmopolitan was co-circulating with DENV-2 genotype Asian-I, the previously predominating genotype in Thailand. Furthermore, DENV-3 genotype III was found instead of DENV-3 genotype II. The DENV-2 Cosmopolitan and DENV-3 genotype III found in Thailand were closely related to the respective strains found in nearby countries. These results indicated that DENVs in Thailand have increased in genotypic diversity, and suggested that the DENV genotypic shift observed in other Asian countries also might be taking place in Thailand.

## Introduction

Dengue virus (DENV) is a mosquito-borne virus that causes various clinical symptoms including dengue fever with and without warning signs and severe dengue, as defined by the 2009 WHO classification [1]. DENV belongs to the *Flavivirus* genus of the family *Flaviviridae* and its genome consists of a single positive-stranded RNA of approximately 11 kb. The DENV genomic RNA has a single open reading frame (ORF) that encodes ten proteins consisting of three structural proteins (C, prM, and E) and seven non-structural proteins (NS1, NS2A, NS2B, NS3, NS4A, NS4B, and NS5). At both the 5’ and 3’ ends, the DENV ORF is flanked by untranslated regions (5’-UTR and 3’-UTR) [2].

There are four distinct serotypes of DENV (DENV-1, DENV-2, DENV-3, and DENV-4) [3]. Within each serotype of DENV, four to six geographically distinct genotypes have been reported. DENV-1 includes genotypes I, II, III (sylvatic), IV, V, and VI [4]. DENV-2 includes Asian-I, Asian-II, Asian/American, American, Cosmopolitan, and sylvatics [5–7]. DENV-3 includes genotypes I, II, III, IV, and V [8]. DENV-4 includes genotypes I, IIA, IIB, III, and sylvatic [8–11]. Thailand is a hyperendemic country where DENVs have been reported for several decades since the 1950s [12]. DENV-3 and DENV-4 were first reported in 1953, while DENV-1 and DENV-2 were documented in 1958 during the first recorded outbreak of dengue hemorrhagic fever [12, 13]. The circulation of the various serotypes is cyclic, with distinct serotypes periodically re-emerging as the dominant serotype. DENV-2 cases were most frequently reported in the 1970s and 1980s. DENV-3 was the major serotype in 1987, 1995–1999, and 2013–2016, while DENV-1 dominated in the 2000s and 2010s. DENV-4 has been detected less frequently, appearing only in 1993–1994 and 2004–2005 [12, 14, 15]. Each of the four serotypes has played an important role as a major serotype in the six outbreaks that have occurred in 1958, 1987, 1998, 2001, 2013, and 2015 [13, 16–20]. It is possible that the introduction of new serotypes or genotypes leads to new epidemics or outbreaks. Until present, the major DENV-2 genotype in Thailand has been Asian-I, which was similar to a genotype detected in other nearby countries in mainland Southeast Asia (notably, Laos and Myanmar). On the other hand, the DENV-2 genotype Cosmopolitan has predominated in other Asian regions, including Maritime South East Asia and South Asia. DENV-2 Asian/American has circulated in the American continents [5, 21–23]. In Thailand, DENV-3 genotype II has been the dominant genotype since DENV-3 was first reported in this country [21, 24–26]. DENV-3 genotype III has spread widely throughout South Asia (notably India, Sri Lanka, and Pakistan), Africa, and the Americas (specifically, Central America, South America, and the Pacific islands) [27–29]. However, DENV-3 genotype III recently was detected in Thailand [30, 31].

In the study presented here, we sought to determine currently circulating DENV serotypes and genotypes in DENV samples obtained at the Bamrasnaradura Infectious Diseases Institute (BIDI) in Thailand during 2016–2017. The nucleotide sequences of near-whole viral genomes were determined by next-generation sequencing followed by phylogenetic analysis of the entire ORF and envelope-encoding region. Here, we show the emergence and circulation of DENV-2 genotype Cosmopolitan and DENV-3 genotype III in Thailand. Our data show recent dynamic changes in DENV epidemiology even in Thailand, a hyperendemic country with multiple DENV serotypes.

## Materials and methods

### Clinical specimens and virus isolation

This study was approved by the Institutional Review Board (IRB) of BIDI (Approved Project Code: S003h/59). Written informed consent was obtained from each participant. One hundred febrile cases presenting at the BIDI with at least one sign indicative of DENV infection (platelet count number less than 100,000 cells/μL; DENV NS1-positive status; or anti-DENV IgM-positive status) were recruited between September 2016 and June 2017. Serum specimens were tested by the DENV-CHIKV multiplex real-time quantitative reverse transcription polymerase chain reaction (RT-PCR) assay (abTESTM DEN/CHIKU 5 qPCR I kit (AITbiotech, Singapore)). Positive sera were diluted with Leibovitz's L-15 medium (Hyclone, USA), filtered through a sterile 0.22-μM membrane, and then inoculated to a confluent layer of *Aedes albopictus*-derived cell line C6/36. After overnight incubation at 28°C, the inocula were removed and replaced with L-15 supplemented with 2% fetal bovine serum (FBS) and 0.3% tryptose phosphate broth [32]. The cells were incubated at 28°C until cytopathogenic effects (CPE) developed without further passages. CPE-positive culture supernatants were tested by real-time RT-PCR specific to DENV [33]. Isolated viruses were further propagated one additional time in Vero cells grown in Minimum Essential Medium (Gibco, USA) supplemented with 2% FBS at 37°C in a 5% CO_2_ environment.

### RNA extraction, reverse transcription, and amplification

Viral RNA was extracted from culture supernatants of Vero cells using a QIAamp viral RNA mini kit (QIAGEN, Germany). Two overlapping cDNA fragments covering the full-length viral genome were synthesized using a SuperScript III first-strand synthesis system (Invitrogen, USA) according to the manufacturer’s instructions. Briefly, 4 μL of extracted viral RNA corresponding to approximately 10^5^–10^7^ copies was mixed either with the previously reported gene-specific sense primer [34, 35] or with a primer designed in the present study (S1 Table). After the reverse transcription, two units of RNaseH were added to each reaction. The resulting cDNA was amplified in a 50-μL PCR reaction containing 5 μL of reverse-transcribed template, 1.25 U of PrimeSTAR GXL DNA polymerase (Takara, Japan), dNTP mixture (200 μM each nucleotide), 0.2 μM each of forward and reverse primers (S1 Table), buffer to a 1x final concentration, and nuclease-free water. The PCR reaction was carried out in an ABI thermal cycler (Applied Bioscience, USA) under the following conditions: 94°C for 1 minute, followed by 35 cycles at 98°C for 10 seconds, 55°C for 30 seconds, and 68°C for 1 minute/kb of expected amplification product. The resulting amplicons were purified with a NucleoSpin gel and PCR clean up kit (MACHEREY-NAGEL, Germany) according to the manufacturer’s instructions, and the quality of the purified DNA product was assessed using a Nanodrop instrument (Thermo Fisher Scientific, USA).

### Library construction and next-generation sequencing

The amplified double-stranded (ds) DNAs were quantified and normalized to 0.2 ng/μL using a Qubit 2.0 fluorometer (Invitrogen, USA). Both the 3’- and 5’-amplicons of each viral isolate were pooled and processed for next-generation sequencing using an Illumina Nextera XT library preparation kit (Illumina, USA) to generate paired-end sequencing libraries. The normalized dsDNA amplicon underwent tagmentation using the transposome provided in the kit. The index 1 (i7), index 2 (i5), and adapters (P5 and P7) required for cluster formation were added to the tagmented DNA using a limited-cycle PCR. The sequencing-ready fragments were purified using Agencourt AMPure XP beads (Beckman Coulter Genomics, USA) and the library concentration was quantified with a Qubit fluorometer. The library size was determined with a high-sensitivity DNA chip processed using a Bioanalyzer 2100 (Agilent Technologies, USA). The library was normalized to 4 nM and libraries with unique indices were mixed to generate pooled libraries.

The pooled libraries were denatured with 0.2 N NaOH and then diluted with HT1 Hybridization buffer (Illumina, USA), to yield library concentrations of 8 pM. phiX (Illumina, USA) was prepared in parallel and spiked into the amplicon libraries at 5% for use as an internal control. The prepared sample was loaded onto a MiSeq v2 kit (500 cycles) reagent cartridge. The paired-end sequencing of 2x 250 bp was processed on the Illumina MiSeq platform, and image analysis and base calling were generated as FASTQ files. During the run, quality was assessed using MiSeq control software. We used sequence data only when more than 75% of the base calls had Q scores over 30.

### Full-length DENV genome assemblies

The FASTQ files were imported into the CLC Genomics Workbench software version 9.5.3 (CLC Bio, QIAGEN) [36]. The forward and reverse reads were aligned to the sequences of DENV-1 Mochizuki (AB074760), DENV-2 16681 (NC 001474), DENV-3 H87 (M93130), and DENV-4 H241 (AY947539) using map-read references, and the consensus sequences were extracted.

### Phylogenetic datasets

DENV sequences obtained in the present study were trimmed to yield ORF regions of final length 10179, 10176, 10173, and 10164 bp for DENV-1, DENV-2, DENV-3, and DENV-4, respectively. For genotype classification, the sequences of the envelope-encoding regions were used so as to include all of the reported genotypes. One hundred and twenty-one DENV envelope-encoding sequences were retrieved from GenBank (http://ncbi.nlm.nih.gov/genbank) based on the previous classification [4, 5, 8–11, 21, 27, 37, 38]. The sequences then were grouped into 4 separate datasets corresponding to each serotype. The DENV-1 genotyping dataset consisted of 27 sequences including 6 distinct genotypes as follows: I (n = 12), II (n = 1), III (n = 1), IV (n = 6), V (n = 6), and VI (n = 1). The DENV-2 genotyping dataset was composed of 41 sequences and included 5 distinct genotypes as follows: Asian-I (n = 9), Asian-II (n = 6), Cosmopolitan (n = 21), American (n = 2), and Asian/American (n = 3). Sylvatic DENV-2 strains were not included in this analysis. The DENV-3 genotyping dataset consisted of 28 sequences, and included 5 distinct genotypes as follows: I (n = 7), II (n = 6), III (n = 6), IV (n = 3), and V (n = 6). The DENV-4 genotyping dataset was composed of 26 sequences, and included 4 distinct genotypes as follows: I (n = 11), II-A (n = 4), II-B (n = 7), and III (n = 4) (S2 Table). Zika virus (AY632535) was used as an outgroup.

Several other datasets were prepared for Bayesian phylogenetic analyses. DENV sequences representing a broad geographical range, from multiple different countries in which DENVs were known to have circulated during the interval from 1975 to 2016, were retrieved from GenBank and combined with sequences obtained in the present study. The sequences of sylvatic strains, laboratory strains, duplicated sequences, and recombinants were excluded from the dataset. All of the sequences were annotated in a format consisting of accession number/country/year of isolation.

The Thailand DENV-2 envelope dataset consisted of 113 DENV-2 sequences reported during 1974–2014, eight sequences obtained in the present study, and one sequence of DENV-2 American genotype as an outgroup (HM582099). The DENV-2 genotype Asian-I envelope dataset was composed of the envelope-encoding sequences obtained in the present study and 92 sequences available in GenBank. The DENV-2 genotype Cosmopolitan envelope dataset was composed of the envelope sequences obtained in the present study and 146 sequences from GenBank. In addition, a DENV-2 genotype Cosmopolitan complete coding sequence dataset was generated by using the sequences obtained in the present study and 47 sequences from GenBank (S3 Table).

A Thailand DENV-3 envelope dataset was generated, including envelope-encoding sequences obtained in the present study along with envelope-encoding sequences from 109 DENV-3 genotype II and 24 DENV-3 genotype III (all of which were DENV-3 isolates that were reported from 1974 to 2016 in Thailand; GenBank), and a DENV-3 genotype-IV sequence (L11433) as an outgroup. Another DENV-3 genotype III envelope dataset was generated including envelope-encoding sequences obtained in the present study and 102 sequences reported from a greater geographical area. In addition, a DENV-3 genotype III complete coding sequence dataset was generated including sequences obtained in the present study and 46 sequences from GenBank (S3 Table).

### Phylogenetic tree inferences and viral evolution analyses

The codon sequences within each dataset were aligned using ClustalW [39] before determination of the substitution model by ModelFinder implemented in IQ-TREE [40]. Phylogenetic trees were inferred from the alignment using the maximum likelihood (ML) approach generated in IQ-TREE [41] with GTR+I+G4 for the DENV genotyping dataset and TN+F+G4 for the Thailand DENV-2 and Thailand DENV-3 datasets with bootstrapping by 500 replicates. For phylogenetic molecular clock analysis, the sequences within the 3 datasets of DENV-2 genotype Asian-I and Cosmopolitan and DENV-3 genotype III were explored using a temporal signal to identify data quality, potential contaminants, and cryptic recombinants by investigating regression of genetic divergence and sampling time by root-to-tip analysis in TempEst [42]. The time of most recent common ancestor (tMRCA) and the rate of evolution (substitution/site/year) were analyzed by BEAST v1.8.4 [43], in which Bayesian Markov Chain Monte Carlo (MCMC) analysis was conducted using the SRD06 substitution model [44]. For selection of the best-fitting model, uncorrelated relaxed log-normal clock (UCLN) and four priors (Constant, Exponential, Bayesian Skyline, and Gaussian Markov random field (GMRF)) were considered. The best-fit demographic and clock model was estimated by path sampling and stepping-stone sampling methods [45]. The MCMC length of chain was run for 40 million-50 million generations with sampling every 4000–5000 generations and 10% burn-in. BEAGLE was used in parallel with BEAST to enhance the running speed [46]. The parameter traces were monitored using Tracer v1.7 [47] with Effective Sample Size values over 200 (ESS >200). The Maximum Clade Credibility (MCC) tree was generated and annotated with posterior probability by TreeAnnotator v1.8.4 [43] and visualized in Figtree. To estimate the history of viral population dynamics, a Gaussian Markov random field (GMRF) Skyride plot was conducted on each of the above 3 datasets using Tracer v1.7. A BEAST log file with rate indicators with asymmetric Bayesian stochastic search variable selection (BSSVS) was conducted for phylogeographic reconstruction in SPREAD v1.0.7 [48] with a Bayes factor cut-off of 3. Obtained results were visualized in Google Earth Pro v7.3.2.5491 (Google Inc., Mountain View, CA, USA) [49]

### Nucleotide and amino acid identity

The coding sequences were analyzed for nucleotide identity by pairwise alignment in the CLC Genomics Workbench software version 9.5.3 [36]. Amino acid identity in the predicted protein sequences also was analyzed.

## Results

### Virus isolation and nucleotide sequence determination of virus genome RNA

Among the 100 febrile cases enrolled in this study, 37 serum specimens tested positive for DENV by DENV-CHIKV multiplex real-time RT-PCR. No cases of CHIKV infection or DENV-CHIKV co-infection were detected. Among these 37 DENV-positive cases, there were 2 DENV-1, 16 DENV-2, 7 DENV-3, and 12 DENV-4 cases. By culturing of the 37 serum specimens that tested positive for DENV, we were able to isolate DENV in C6/36 from 21 serum specimens. There was a wide variation in levels of serum DENV measured by the real-time quantitative RT-PCR, but virus was more readily isolated from specimens with higher DENV loads since mean Ct value for isolation-positive specimens was 22.4 while that of isolation-negative specimens was 31.5 (P<0.0001, t-test). The obtained virus isolates then were propagated in Vero cells. These isolates included 1 DENV-1, 8 DENV-2, 4 DENV-3, and 8 DENV-4. Nucleotide sequences of near-full-length virus genomes (10604–10694 nucleotides) were successfully determined with Quality Score 64 and submitted to GenBank (accession numbers LC410183-LC410203) (Table 1). ORF lengths of 10179, 10176, 10173, and 10164 bp were obtained for DENV-1, -2, -3, and -4, respectively. The coding sequences of the 8 DENV-2 isolates obtained in the present study exhibited wide variations in sequence similarity, with 90.55% - 99.92% and 97.11% - 99.94% identity at the nucleotide and amino acid levels, respectively. Among 8 DENV-2 isolates, 5 isolates (Th16-005DV2, Th16-035DV2, Th16-056DV2, Th17-061DV2, and Th17-074DV2) showed 97.65% - 99.92% and 99.29–99.94% identity at the nucleotide and amino acid levels, respectively. Similarly, the 3 remaining DENV-2 isolates (Th16-011DV2, Th16-026DV2, and Th17-077DV2) showed high levels of identity, with values of 99.78% and 99.91–99.04% at the nucleotide and amino acid levels, respectively. These results suggested that two distinct genotypes or lineages of DENV-2 were observed within this data set. On the other hand, the 4 DENV-3 sequences showed nucleotide and amino acid identities of 99.44% - 99.97% and 99.71% - 99.97%, respectively. The nucleotide and amino acid identities of the 8 DENV-4 sequences were 98.22% - 99.99% and 99.26% - 100%, respectively.

**Table 1:** Dengue virus isolates characterized in the present study.

Isolate	Area	Collection date	Serotype	Genotype	Passage History	Accession no.
Th17-087DV1	Chiang Mai	April, 2017	1	I	C6/36, Vero	LC410183
Th16-005DV2	Ratchaburi	September, 2016	2	Asian-I	C6/36, Vero	LC410184
Th16-035DV2	Nonthaburi	November, 2016	2	Asian-I	C6/36, Vero	LC410185
Th16-056DV2	Nonthaburi	December, 2016	2	Asian-I	C6/36, Vero	LC410186
Th17-061DV2	Khaoyai	January, 2017	2	Asian-I	C6/36, Vero	LC410187
Th17-074DV2	Nonthaburi	February, 2017	2	Asian-I	C6/36, Vero	LC410188
Th16-011DV2	Nonthaburi	October, 2016	2	Cosmopolitan	C6/36, Vero	LC410189
Th16-026DV2	Uttaradit	October, 2016	2	Cosmopolitan	C6/36, Vero	LC410190
Th17-077DV2	Nonthaburi	February, 2017	2	Cosmopolitan	C6/36, Vero	LC410191
Th16-016DV3	Nonthaburi	October, 2016	3	III	C6/36, Vero	LC410192
Th16-049DV3	Phetchabun	December, 2016	3	III	C6/36, Vero	LC410193
Th16-055DV3	Nonthaburi	December, 2016	3	III	C6/36, Vero	LC410194
Th17-059DV3	Nonthaburi	January, 2017	3	III	C6/36, Vero	LC410195
Th16-001DV4	Pathum Thani	September, 2016	4	I	C6/36, Vero	LC410196
Th16-006DV4	Nonthaburi	September, 2016	4	I	C6/36, Vero	LC410197
Th16-010DV4	Nonthaburi	October, 2016	4	I	C6/36, Vero	LC410198
Th16-020DV4	Nonthaburi	October, 2016	4	I	C6/36, Vero	LC410199
Th16-037DV4	Nonthaburi	November, 2016	4	I	C6/36, Vero	LC410200
Th16-046DV4	Nonthaburi	December, 2016	4	I	C6/36, Vero	LC410201
Th17-091DV4	Nonthaburi	June, 2017	4	I	C6/36, Vero	LC410202
Th17-098DV4	Nonthaburi	June, 2017	4	I	C6/36, Vero	LC410203

### Emergence of the novel DENV-2 and DENV-3 genotypes in Thailand during 2016–2017

**Fig 1:**
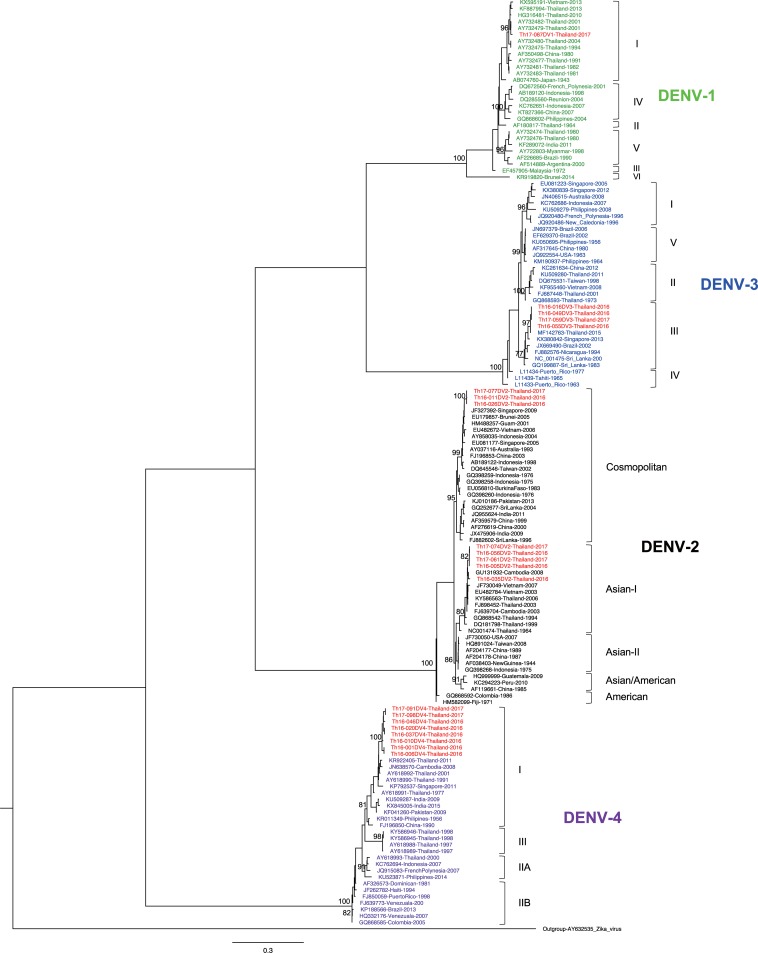
Dengue virus genotyping. The maximum likelihood phylogenetic tree was constructed in the IQ-TREE program using the general time reversible model with gamma distribution and invariant sites with 500 bootstrap replications. Data included envelope-encoding sequences obtained in the present study (labeled in red) along with sequences obtained from GenBank. The viral genotypes are indicated to the right. Virus names are shown as accession number, country, and reported year of each sequence. Numbers on branches are bootstrap support values exceeding 75%.

To determine the genotype of DENV obtained in the present study, ML trees were generated. The resulting ML tree showed that the DENV-1 isolate obtained in the present study (Th17-087DV1) belonged to genotype I (Fig 1), the major genotype circulating in Thailand [24]. In the case of DENV-2, the phylogenetic analysis revealed the existence of 2 co-circulating genotypes in Thailand during 2016–2017. Five isolates (Th16-005DV2, Th16-035DV2, Th16-056DV2, Th17-061DV2, and Th17-074DV2) were genotyped as Asian-I, a local genotype. On the other hand, the other 3 isolates (Th16-011DV2, Th16-026DV2, and Th17-077DV2) belonged to genotype Cosmopolitan, a genotype known to be circulating in Southeast Asia that (previously) had been found only rarely and sporadically in Thailand (Fig 1) [5, 21]. The patients harboring DENV-2 genotype Cosmopolitan came to the hospital from Nonthaburi (Central Thailand) or Uttaradit (Northern Thailand, approximately 350 km from Nonthaburi) on separate dates (Table 1). For DENV-3, the ML tree revealed that all 4 of the isolates that we obtained (Th16-016DV3, Th16-049DV3, Th16-055DV3, and Th17-059DV3) were classified as genotype III (Fig 1), although genotype II has previously been considered the major genotype in Thailand [24]. Finally, all 8 of the DENV-4 isolates that we obtained belonged to genotype I (Fig 1), the major genotype circulating in Thailand [10].

These phylogenetic analyses showed the presence of DENV-2 genotype Cosmopolitan and DENV-3 genotype III in Thailand during 2016–2017, although these genotypes have been detected only rarely in this country. As of the writing of this paper, no full ORF sequence of DENV-2 genotype Cosmopolitan in Thailand previously was available in GenBank. Only one full ORF sequence of DENV-3 genotype III in Thailand was available in GenBank, although details were not disclosed (MF14273). These results suggest an increase in DENV genotypic diversity in Thailand.

### Time of introduction of the newly emerged DENV genotypes in Thailand

**Fig 2:**
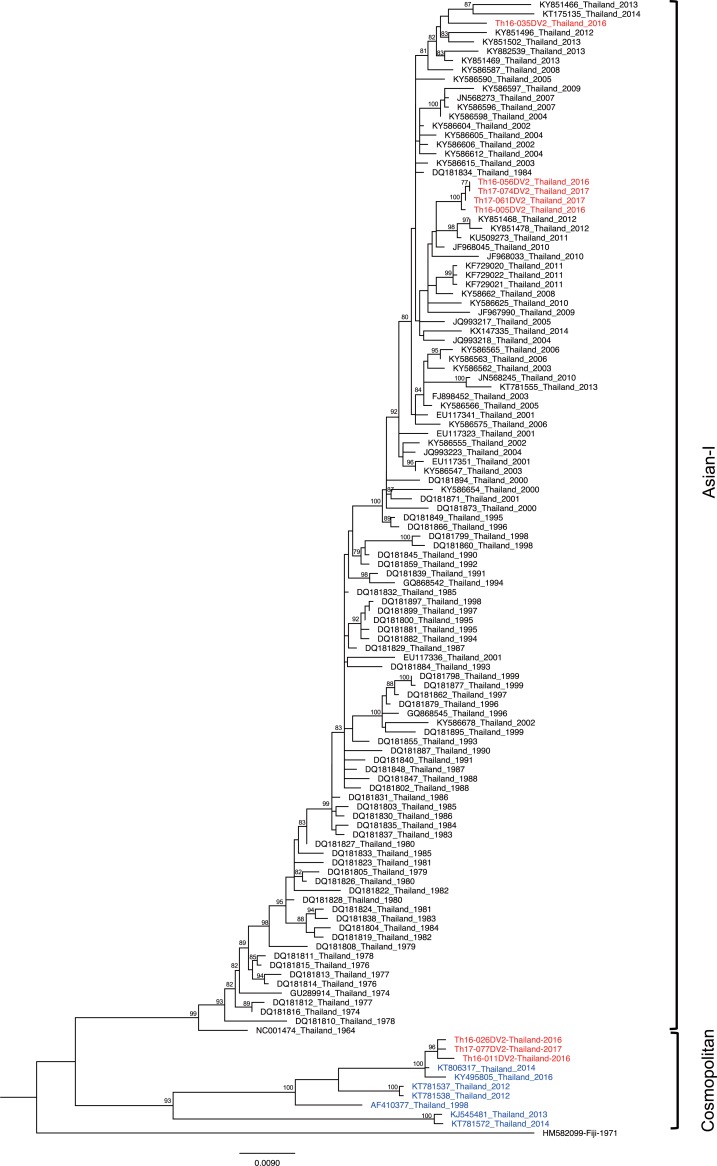
Phylogeny of Thailand DENV-2 strains. The maximum-likelihood phylogenetic tree was constructed in IQ-TREE version 1.6.7 using the TN+F+G4 with 500 bootstrap replications. Data included envelope-encoding sequences obtained in the present study (labeled in red) along with sequences obtained from Genbank (Asian-I, black; Cosmopolitan, blue). The viral genotypes are indicated to the right. Virus names are shown as accession number, country, and reported year of each sequence. Numbers on branches are bootstrap support values exceeding 75%.

**Fig 3:**
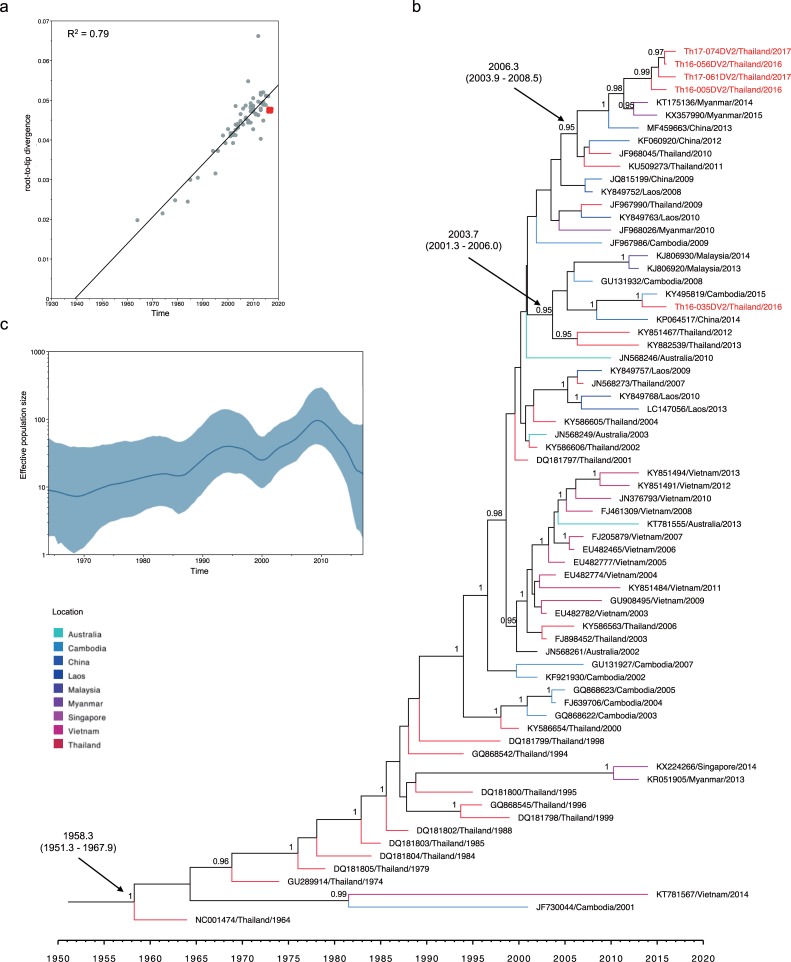
Molecular clock analysis of DENV-2 genotype Asian-I envelope-encoding sequences. (a) Correlation of collection year and divergence from the maximum likelihood tree. The R2 (coefficient of determination) of 0.79 was estimated using TempEst (shown at the top left). Red dots indicate sequences obtained in the present study. (b) Bayesian maximum clade credibility (MCC) phylogenetic tree estimated using BEAST v1.8.4. The terminal branch color indicates different geographical locations according to the legend at the bottom left corner of the figure. The mean time of the most recent common ancestor (tMRCA) and 95% highest probability density (HPD) (in calendar year and tenths of year) are indicated with a black arrow, and posterior probability values are indicated adjacent to the node of interest. The name of each taxon is presented formatted as accession number, country, and year of collection. Sequences obtained in the present study are labeled in red. (c) Demographic history of DENV-2 genotype Asian-I was inferred by a Gaussian Markov random field (GMRF) Skyride plot using Tracer 1.7.

**Fig 4:**
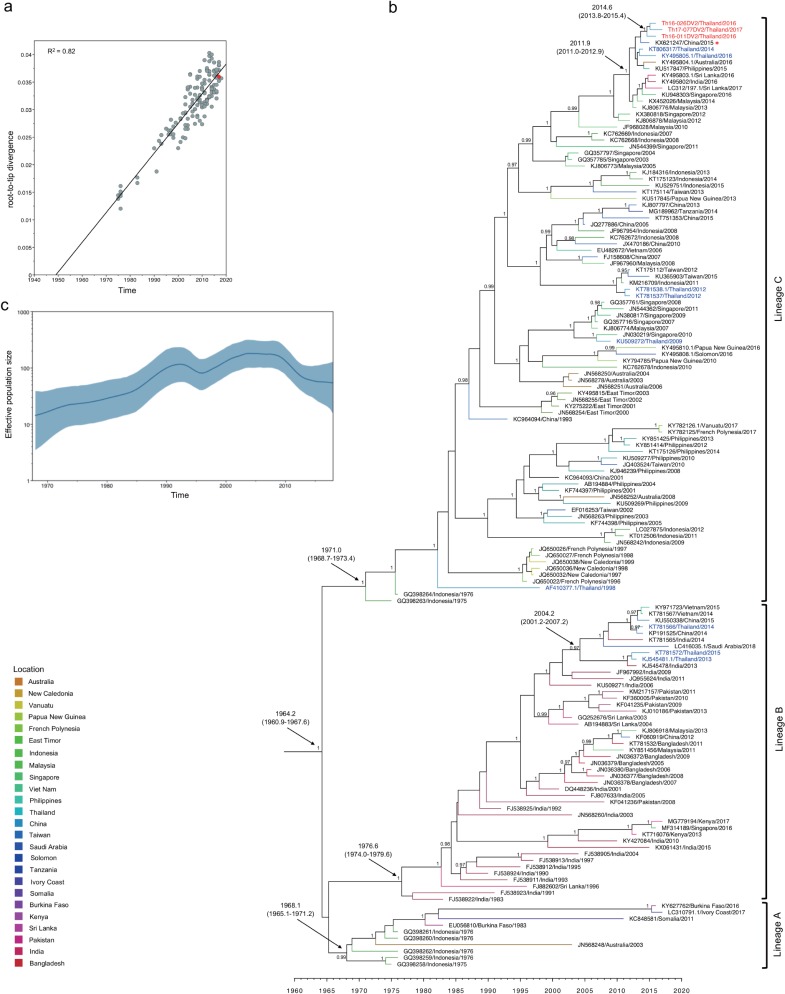
Molecular clock analysis of DENV-2 genotype Cosmopolitan envelope-encoding sequences. (a) Correlation of collection year and divergence from maximum likelihood tree. The R^2^, (coefficient of determination) of 0.82 was estimated using TempEst (shown at the top left). Red dots indicate sequences obtained in the present study. (b) Bayesian maximum clade credibility (MCC) phylogenetic tree estimated using BEAST v1.8.4. The terminal branch color indicates different geographical locations according to legend at the bottom left corner of the figure. The mean time of the most recent common ancestor (tMRCA) and 95% highest probability density (HPD) (in calendar year and tenths of year) are indicated with black arrows, and posterior probability values are indicated adjacent to the node of interest. The name of each taxon is presented formatted as accession number, country, and year of collection. Sequences obtained in the present study are labeled in red, while Thailand sequences obtained from GenBank are labeled in blue. Lineages A, B, and C are shown to the right. The KX621247 virus is indicated by a red asterisk. (c) Demographic history of DENV-2 genotype Cosmopolitan was inferred by a Gaussian Markov random field (GMRF) Skyride plot using Tracer 1.7.

Previously, DENV-2 genotype Cosmopolitan was detected in Maritime South East Asia (Indonesia, Malaysia, Singapore, and Philippines) [50–54] and South Asia (India, Bangladesh, Nepal, Pakistan, and Sri Lanka) [5, 6, 55, 56]. The phylogenetic tree of the envelope genes of DENV-2 Thailand strains collected from 1964–2017 confirmed the presence of two genotypes in separate monophyletic clusters (Fig 2). The Asian-I cluster consisted of viruses observed during 1964–2017, whereas viruses within the Cosmopolitan cluster first appeared in 1998 and were detected only intermittently until 2017 (Fig 2). The co-circulation of 2 genotypes of DENV-2, a local genotype of Asian-I and Cosmopolitan prompted us to attempt an estimation of the time of introduction by molecular clock analysis. For this purpose, we prepared another two datasets of envelope-encoding sequences of DENV-2 genotype Asian-I and DENV-2 genotype Cosmopolitan. These datasets were composed of DENV-2 sequences obtained in the present study along with the other DENV-2 sequences recovered from various DENV-endemic countries between 1975 and 2016. Root-to-tip regression analyses of these new datasets showed a high degree of correlation (R^2^ = 0.79–0.82) (Figs 3A and 4A) between collection year and divergence, suggesting appropriate samplings for the molecular clock analysis.

The time-scaled Bayesian phylogenetic tree based on envelope-encoding sequences (constructed using UCLN and Bayesian Skyline coalescent prior, which was the best-fitting model (S4 Table)) (Fig 3B) revealed that the DENV-2 Asian-I isolates (Th16-005DV2, Th16-056DV2, Th17-061DV2, and Th17-074DV2) clustered together with recent viral strains from Thailand, Myanmar, and China (posterior probability (PP) of 0.95), with a tMRCA of 2006.3 (95% HPD: 2003.9–2008.5). Isolate Th16-035DV2 was closely related to Malaysia, Cambodia, and China viruses (PP of 0.95), yielding a tMRCA of 2003.7 (95% HPD: 2001.3–2006.0). On the other hand, DENV-2 Asian-I yielded an estimated mean tMRCA of 1958.3 (95% HPD: 1951.3–1967.9) and an estimated mean substitution rate of 1.04 x 10^−3^ substitutions per site per year (95% HPD: 8.27 x 10^−4^–1.27 x 10^−4^ substitutions per site per year). The oldest DENV-2 Asian-I isolate, which had been reported in 1964 in Thailand, served as the root of this tree [57]. Subsequently, the Asian-I genotype viruses circulated throughout mainland Southeast Asia, including Thailand. The GMRF Skyride plot of the DENV-2 Asian-I sequences in Fig 3C showed that this genotype exhibited a slight increase in effective population size (Ne) during 1987–1995. The Ne then increased significantly between 2000–2010. These increases occurred concurrently with the introduction of Asian-I into other countries (Cambodia and Vietnam) that are close to Thailand [15, 58–60].

As shown in Fig 4B, Bayesian phylogenetic analysis using the DENV-2 Cosmopolitan envelope dataset (constructed using the UCLN and GMRF coalescent prior as the best-fitting model (S4 Table)) showed that the tree was rooted at 1964.2 (95% HPD: 1960.9–1967.6). The mean substitution rate was 8.81 x 10^−4^ substitutions per site per year (95% HPD: 7.76 x 10^−4^–9.89 x 10^−4^ substitutions per site per year). The phylogenetic tree comprised 3 lineages with posterior probabilities above 0.99 (designated as lineages A, B, and C). The tMRCA of lineage A was estimated as 1968.1 (95% HPD: 1965.1–1971.2), and viral strains within this lineage were detected in Indonesia in 1975–1976 and in Burkina Faso in 1983, 1986, and (most recently) 2016. Lineage B contained viruses isolated in South Asia (i.e., India, Pakistan, Bangladesh and Sri Lanka) as well as China and Saudi Arabia. Viruses of lineage B shared a tMRCA of 1976.6 (95% HPD: 1974.0–1979.6) while those of lineage C showed a similar tMRCA of 1971.0 (95% HPD: 1968.7–1973.4). However, lineage C contained viral strains that predominated in Southeast Asia (i.e., Indonesia, Philippines, Singapore, and Malaysia), the Pacific islands (i.e., Papua New Guinea and New Caledonia), and Australia. The Cosmopolitan viruses obtained in the present study clustered within lineage C. The tMRCA of these viruses was estimated as 2014.6 (95% HPD: 2013.8–2015.4). Two DENV-2 Cosmopolitan isolates, Th16-011DV2 and Th16-026DV2, were obtained in October 2016, while Cosmopolitan isolate Th17-077DV2 was collected in February 2017. None of the three patients from whom these viruses were recovered had a travel history before the onset of fever, suggesting that DENV-2 Cosmopolitan had been circulating in Thailand. Two similar strains, which were obtained from Thai travelers in Australia, have been reported (KY495805 and KT806317) [61]. These 5 recent genotype Cosmopolitan viruses shared a tMRCA with other viruses isolated in 2012–2017 (specifically in China, the Philippines, Australia, Sri Lanka, India, Malaysia, and Singapore) of 2011.9 (95% HPD: 2011.0–2012.9). Two distantly related lineage-C viruses and two lineage-B viruses also were obtained from Thai travelers in Australia [61]. These lineage-B viruses were clustered with viruses isolated in Viet Nam, China, Saudi Arabia, and India. Finally, a more distantly related Thailand lineage-C virus was obtained in 1998 (AF410377.1) [5].

The GMRF Skyride plot in Fig 4C suggests that DENV-2 Cosmopolitan viruses also appear to have experienced two phases of exponential increase of Ne during 1965–1992 and 1996–2002. This inference is consistent with the fact that during the 2000s, DENV-2 Cosmopolitan viruses were introduced into several regions in Maritime Southeast Asia (Indonesia, Malaysia, and the Philippines) [51–53] and into regions of South Asia (India, Pakistan, and Sri Lanka) [5, 28, 62].

The visualizing MCC tree reconstruction based on phylogeographic analysis of the DENV-2 genotype Cosmopolitan shows several possible routes of viral introduction into Thailand. The source for these possible routes were China, India, Singapore, Malaysia, and Indonesia (S1 Fig). Among these sources, China seems the most likely for explaining the origin of DENV-2 Cosmopolitan viruses obtained in the present study (Fig 4B, S1 Fig, and S3 Fig), since a virus obtained in China in 2015 (KX621247) was most closely related to these viruses both in the envelope-coding region (Fig 4B) and entire ORF (S3 Fig).

**Fig 5:**
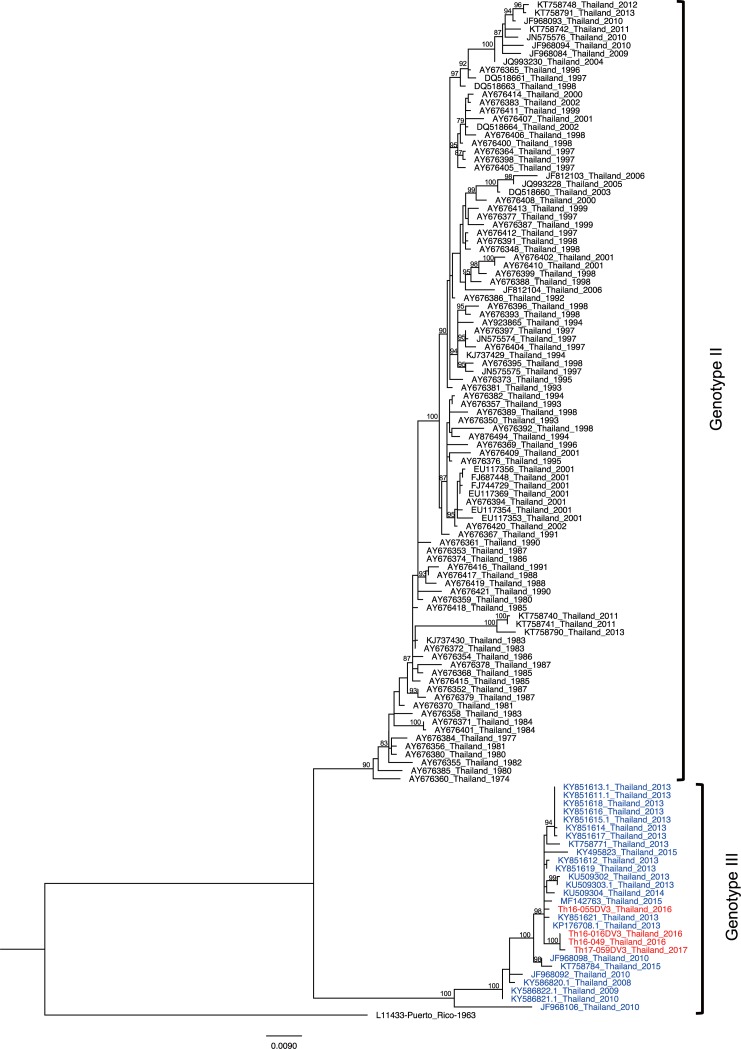
Phylogeny of Thailand DENV-3 strains. The maximum likelihood phylogenetic tree was constructed in IQ-TREE version 1.6.7 using the TN+F+G4 with 500 bootstrap replications. Data included envelope-encoding sequences obtained in the present study (labeled in red) along with sequences obtained from Genbank (genotype II, black; genotype III, blue). The viral genotypes are indicated to the right. Virus names are shown as accession number, country, and reported year of each sequence. Numbers on branches are bootstrap support values exceeding 75%.

**Fig 6:**
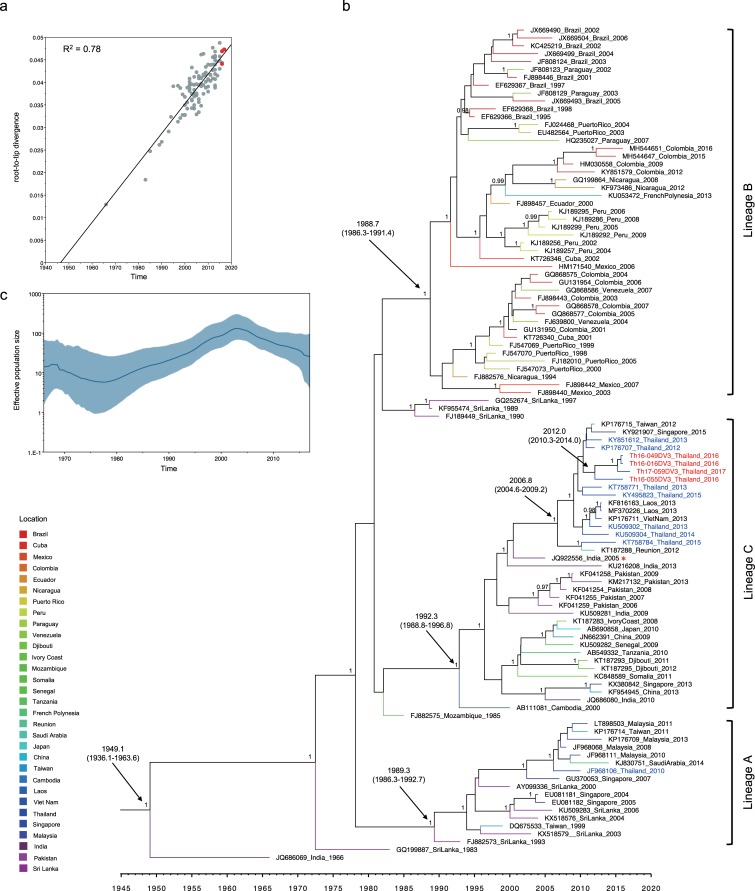
**Molecular clock analysis of DENV-3 genotype III envelope-encoding sequences** (a) Correlation of collection year and divergence from maximum likelihood tree. The R^2^ (coefficient of determination) of 0.78 was estimated using TempEst (shown at top left). Red dots indicate sequences obtained in the present study. (b) Bayesian maximum clade credibility (MCC) phylogenetic tree estimated using BEAST v1.8.4. The terminal branch color indicates different geographical locations according to the legend at the bottom left corner of the figure. The mean time of the most recent common ancestor (tMRCA) and 95% highest probability density (HPD) (in calendar year and tenths of year) are indicated with black arrows, and posterior probability values are indicated adjacent to the node of interest. The name of each taxon is presented formatted as accession number, country, and year of collection. Sequences obtained in the present study are labeled in red, while Thailand sequences from GenBank are labeled in blue. Lineages A, B, and C are shown to the right. The JQ922556 virus is indicated by a red asterisk. (c) Demographic history of DENV-3 genotype III was inferred by a Gaussian Markov random field (GMRF) Skyride plot using Tracer 1.7.

DENV-3 viruses obtained in the present study carried sequences characteristic of genotype III, not those characteristic of genotype II, even though genotype II has constituted the dominant genotype reported in Thailand since 1973 [5, 22, 60]. The phylogenetic tree of the envelope-encoding sequences of DENV-3 strains collected in Thailand from 1974 to 2017 confirmed the presence of the two genotypes in separate monophyletic clusters (Fig 5). The genotype-II cluster consisted of viruses collected from 1974 to 2013, whereas viruses within the genotype-III cluster were first observed in 2008 and subsequently continued to be detected until 2017 (Fig 5). To investigate the time of introduction of DENV-3 genotype III into Thailand, the DENV-3 genotype III dataset was constructed; this dataset showed a high degree of correlation (R^2^ = 0.78) between collection year and divergence (Fig 6A), suggesting that these data constituted an appropriate sampling. Bayesian phylogenetic analysis (constructed using UCLN and Bayesian Skyline coalescent prior, which was the best-fitting model (S4 Table)) (Fig 6B) showed that the root of the DENV-3 genotype III tree was estimated as 1949.1 (95% HPD: 1936.1–1963.6). The mean substitution rate was 6.74 x 10^−4^ substitutions per site per year (95% HPD: 5.45 x 10^−4^–8.09 x 10^−4^ substitutions per site per year). The phylogenetic tree showed 3 lineages (posterior probability = 1). Lineage A contained viruses isolated from Sri Lanka, Singapore, Malaysia, Saudi Arabia, and Taiwan. Lineage B consisted of viral strains circulating in the South American region (Brazil, Paraguay, Peru, Nicaragua, Colombia, and Cuba) and the Pacific islands. Lineage C comprised viruses isolated from South Asia (Pakistan and India) and Southeast Asia (Laos and Viet Nam). The tMRCAs of each of the lineages were estimated as 1989.3 (95% HPD: 1986.3–1992.7), 1988.7 (95% HPD: 1986.3–1991.4), and 1992.3 (95% HPD: 1988.8–1996.8), respectively. Th16-016DV3, Th16-049DV3, Th16-055DV3, and Th17-059DV3 clustered within lineage C and appeared to be closely related to viruses from Thailand, Singapore, Laos, and Vietnam. The tMRCA of DENV-3 genotype III isolates obtained in Thailand from 2015 to 2017 was 2006.8 (95% HPD: 2004.6–2009.2), while the tMRCA of viruses that we obtained (Th16-016DV3, Th16-049DV3, Th16-055DV3, and Th17-059DV3) was 2012.0 (95% HPD: 2010.3–2014.0). The DENV-3 genotype III Ne was inferred to exhibit an exponential growth phase from 1980 to 2003, when viruses achieved a maximum number (Fig 6C), consistent with virus dissemination in multiple countries in multiple regions [23, 27, 29, 63].

The visualizing MCC tree reconstruction based on phylogeographic analysis of the DENV-3 genotypes III shows that India and Malaysia were two possible routes of viral introduction into Thailand (S2 Fig). DENV-3 genotypes III isolated from our study share a common ancestor with Indian strains (JQ922556 and KU216208); therefore, we infer that the route from India is most likely the origin of the DENV-3 genotype III viruses obtained in the present study (Fig 6B, S2 Fig, and S4 Fig).

### Molecular signatures of newly introduced DENV genotypes

**Fig 7:**
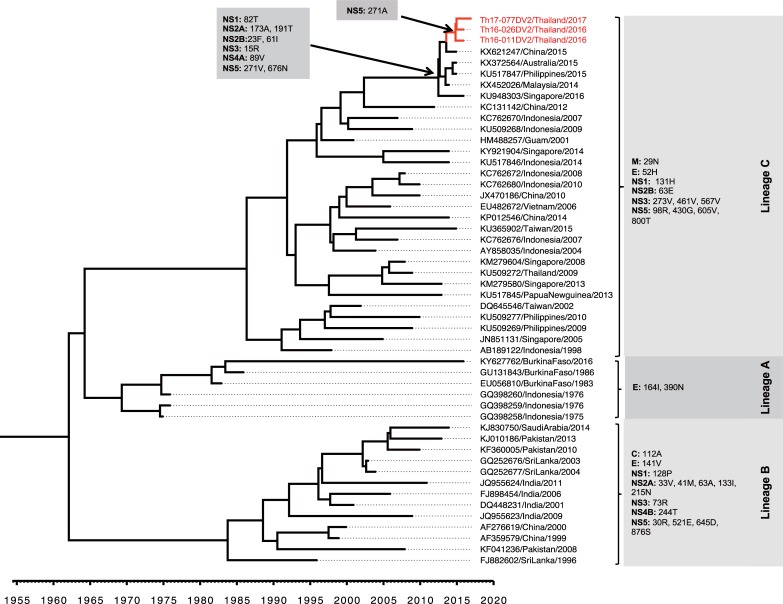
Amino acid variations among ORF proteins encoded by DENV-2 genotype Cosmopolitan. The amino acid substitutions in the open reading frames of the 3 lineages of DENV-2 genotype Cosmopolitan (in the right panel) are shown corresponding to the lineages. The occurrences of indicated amino acid variations are shown with arrows. Sequences obtained in the present study are labeled in red.

The variation of amino acid substitution among DENV-2 Cosmopolitan was analyzed based on the complete coding sequences. The presence of 36 amino acid variations was detected among the 3 lineages of DENV-2 Cosmopolitan according to lineages in the corresponding phylogenetic tree (Fig 7). In particularly, Th16-011DV2, Th16-026DV2, and Th17-077DV2 shared 11 amino acid residues (i.e., 29N in protein M; 52H in protein E; 131H in protein NS1; 63E in protein NS2B; 273V, 461V and 567V in protein NS3; and 98R, 430G, 605V, and 800T in protein NS5) with other lineage-C viruses, and shared 8 more amino acid substitutions (i.e., 82T in protein NS1; 173A and 191T in protein NS2A; 23F and 61I in protein NS2B; 15R in protein NS3; 89V in protein NS4A; and 676N in protein NS5) in common with recent lineage-C isolates reported after 2014, substitutions that were not observed in lineage-A or lineage-B viruses. Notably, the 271A substitution in protein NS5 was found only in the 3 Cosmopolitan isolates obtained in the present study.

**Fig 8:**
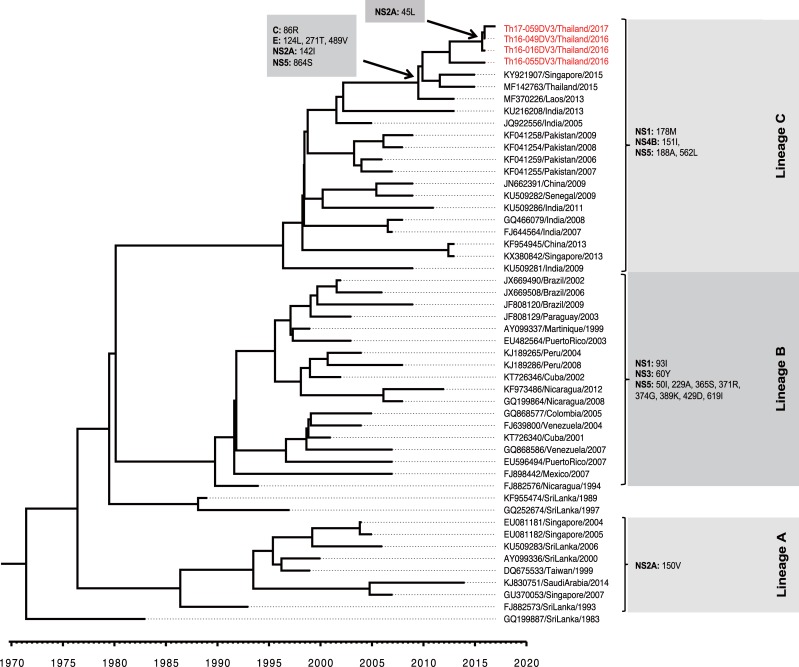
Amino acid variations among ORF proteins encoded by DENV-3 genotype III. The amino acid substitutions in the open reading frame among 3 lineages of DENV-3 genotype III (in the right panel) are shown corresponding to the lineages. The occurrences of the indicated amino acid variations are shown with arrows. Sequences obtained in this study are labeled in red.

On the other hand, 21 amino acid variations were detected among the 3 lineages of DENV-3 genotype III viruses. Among these, four amino acid residues (i.e., 178M in protein NS1; 151I in protein NS4B; and 188A, and 562L in protein NS5) were present in the isolates obtained in the present study and were shared with the South Asia lineage C (including viruses found in India and Pakistan). In addition, 6 amino acid residues (i.e., 86R in protein C; 124L, 271T, and 489V in protein E; 142I in protein NS2A; and 864S in protein NS5) were shared among isolates obtained in Thailand, Singapore, and Laos during the 2013–2017 interval. Only the 45L substitution in protein NS2A was found to be present in all of the DENV-3 genotype III viruses obtained in the present study (Fig 8).

## Discussion

In the present study, we determined nearly complete nucleotide sequences of DENV isolated from 21 DENV-positive patients at the Bamrasnaradura Infectious Diseases Institute in Nonthaburi province, a region close to Bangkok, the capital city of Thailand, during the period of September 2016 to June 2017. To minimize possible mutations during the isolation procedure, we passaged our isolates only once in Vero cells before sequencing. We found examples of all four DENV serotypes in less than one year. Notably, we identified more than one case each of DENV-2 genotype Cosmopolitan and DENV-3 genotype III, both of which previously have only rarely been reported in Thailand. These results suggested that DENV-2 genotype Cosmopolitan and DENV-3 genotype III were emerging virus genotypes in recent years in Thailand.

Among eight DENV-2 isolates, there were three genotype Cosmopolitan viruses. Although sequence analysis revealed that these three DENV-2 isolates were most closely related to each other, the three cases visited the hospital at different times, and one patient lived at a distance of approximately 350 km from the hospital. Therefore, it is highly likely that DENV-2 genotype Cosmopolitan has started to circulate within Thailand. Based on 1485 bp of nucleotide sequences encoding the DENV envelope protein, we estimated that these three isolates originated from a common ancestor in 2014. On the other hand, the estimated tMRCA of 1964 (95% HPD: 1960.9–1967.6) of DENV-2 genotype Cosmopolitan in our dataset was in a good agreement with previous findings that estimated the DENV-2 Cosmopolitan tMRCA as having occurred in the 1950s to 1960s [7, 64, 65], confirming that our method of estimation was valid. When we used the entire ORF sequences of 10176 bp, the tMRCA of the three DENV-2 Cosmopolitan viruses obtained in the present study was 2014.8 (95% HPD: 2014.4–2015.3) (S3 Fig), which is almost identical to the value based on the envelope-encoding sequences (2014.6 (95% HPD: 2013.7–2015.4)). The mean substitution rate of DENV-2 Cosmopolitan, 8.81 x 10^−4^ substitutions per site per year, obtained in the present study, was slightly lower than the value of 6.20 x 10^−4^ substitutions per site per year (95% HPD: 4.00 x 10^−4^–8.45 x 10^−4^ substitutions per site per year) obtained in a previous study [65]. The overall median substitution rate for DENV-2 viruses was estimated to be 6.98 x 10^−4^ substitutions per site per year (95% HPD: 5.00 x 10^−4^–8.94 x 10^−4^ substitutions per site per year) [66]. However, slightly different substitution rates have been observed among different genotypes, including values (in substitutions per site per year) of 1.05 x 10^−4^ (95% HPD: 8.27 x 10^−4^–9.96 x 10^−4^) for Asian-I in the present study, and 7.82 x 10^−4^–1.26 x 10^−3^ for Asian-American and 6.54 x 10^−4^ (95% HPD: 4.61 x 10^−4^–8.92 x 10^−4^) for American in previous studies [65, 67].

Although DENV-2 Cosmopolitan viruses have circulated in Asia for at least 55 years, viruses of this genotype were restricted to countries of South Asia and Maritime Southeast Asia. To date, only a few papers (to our knowledge) have reported the presence of the DENV-2 genotype Cosmopolitan in Thailand. One such paper reported, in 2009, that a German traveler had acquired a DENV-2 infection in Thailand [68]. Phylogenetic analysis of the DENV-2 genotype Cosmopolitan viruses obtained in the present study suggested a branch time for DENV-2 genotype Cosmopolitan of late 2014. Analysis of the envelope-encoding sequences, as well as that of the entire ORF sequence, yielded consistent results suggesting that DENV-2 genotype Cosmopolitan viruses obtained in the present study clustered with lineage-C viruses and were closely related to recently isolated viruses from China, the Philippines, Malaysia, Singapore, and Australia, areas in which Cosmopolitan was the local genotype. A distinct subset of eight amino acid residues in the complete coding sequence (shown in Fig 7) was shared among these viruses; the 271A substitution located in protein NS5 was characteristic of the isolates in the present study. This substitution occurred at a site (amino acid 271) that is located in an interdomain linker known to play an important role in viral replication [69]. Interestingly, nine of the DENV-2 genotype Cosmopolitan isolates were detected in southern China, a location that is near Laos and northern Thailand. Farther from Thailand (i.e., on the other side of southern China (Yunnan Province), closer to the border with Myanmar), only the Asian-I genotype has been detected [70].

Phylogeographic analysis of the DENV-2 Cosmopolitan viruses suggested a route from China might explain the origin of our Thailand DENV-2 Cosmopolitan viruses obtained in the present study (Fig 4B, S1 Fig, and S3 Fig). However, this possibility will need to be further evaluated in future research.

Among the eight new DENV-2 isolates, the remaining (non-Cosmopolitan) five were genotype Asian-I. Therefore, the co-circulation of DENV-2 Asian-I and Cosmopolitan was observed in 2016 and 2017 in Thailand. It will be important to see whether Asian-I is replaced by Cosmopolitan in subsequent years, given that Asian-I itself completely replaced Asian/American in Vietnam and Cambodia in 2008 and 2005, respectively [58]. If genotype replacement does occur, it will be important to see whether existing diagnostic measures work as well for the new genotype as those measures did for the previous genotype, whether the new genotype causes a larger outbreak than the previous one, and whether the disease severity changes. However, the genotype replacement of DENV may not necessarily follow automatically after the introduction of the new lineage; indeed there is precedent (in Brazil) for the failure of a newly introduced DENV-1 lineage to replace a pre-existing lineage of DENV-1 [71]. Our phylogeographic analysis also suggested repeated introduction of DENV-2 genotype Cosmopolitan into Thailand without detectable outbreaks. Therefore, it also will be interesting to compare the replicative capabilities and antigenicities of the new isolates of DENV-2 genotype Cosmopolitan and Asian-I viruses.

A review of the history of DENV-3 in Thailand revealed that three isolates of DENV-3 genotype III were observed (among 123 DENV-3 isolates) during 2008–2010 [72]. The DENV-3 genotypes II and III (3 genotype II and 3 genotype III viruses) were detected in 6 Taiwanese patients; these events were classified as imported cases by the Centers for Diseases Control, Taiwan, based on the fact that these individuals had a history of travel to Thailand before seeking medical care [30]. Interestingly in the period of 2011–2013, a total of 11 DENV-3 cases was identified from 71 confirmed DENV cases in Thailand and classified as genotype III infections [31]. Furthermore, our comprehensive phylogenetic analysis based on the envelope-encoding sequences of viruses collected in Thailand (Fig 5) revealed that genotype III has predominated since 2010, and the most recent DENV-3 genotype II viruses in Thailand were obtained in 2013. These lines of evidence suggest that a genotypic shift occurred in Thailand, such that DENV-3 genotype II, a local genotype maintained in Thailand since 1973, was replaced with genotype III. However, we are unable to exclude the possibility of an ongoing, small-scale local circulation of DENV-3 genotype II in Thailand.

In the present study, the DENV-3 genotype III tMRCA was estimated (based on envelope-encoding sequences) to be 1949 (95% HPD: 1936.1–1963.6), consistent with the previous study [73]. However, this value (again, obtained based on envelope-encoding sequences) differed from the tMRCA of 1967 (95% HPD: 1965–1969) reported by Tan et al. [74] based on analysis of the complete viral coding sequence. This difference might be due to the fact that the oldest virus in our analysis was from 1966 (JQ868069) and represented an isolate from which only the envelope sequence was available. Furthermore, in the present study, the substitution rate of DENV-3 genotype III was 6.74 x 10^−4^ substitutions per site per year, whereas the substitution rate of viruses in the Americas has been estimated to fall in the range of 8.2 x 10−4–1.03 10^−3^ substitutions per site per year [75].

The introduction of DENV-3 genotype III into Thailand was estimated to have occurred before 2006, since the tMRCA of Thailand DENV-3 genotype III viruses was estimated as 2006.8 (95% HPD: 2004.6–2009.2) based on envelope-encoding sequences. We obtained a similar tMRCA value of 2009.9 (95% HPD: 2008.8–2011.1) when we analyzed the entire 10173-bp ORF sequence (S4 Fig). It will be important to investigate whether the newly emerged DENV-3 genotype III led to the 2013 and 2015 DENV outbreaks in Thailand, since DENV-3 was the dominant serotype, accounting for 36.59% and 33.30% of DENV cases in 2013 and 2015, respectively [16, 17]. Outside of Thailand, DENV-3 genotype III also has been disseminated to other countries in Asia. DENV-3 genotype III was isolated as a minor genotype in Laos in 2012–2013 [76], and was first observed in Vietnam in 2013 [77], in Singapore in 2013 [78], and in Malaysia in 2008–2010 [74]. Our study revealed that the DENV-3 genotype III currently circulating in Thailand clusters with lineage-C viruses from South Asia (India and Pakistan) and from other areas in Southeast Asia (Singapore, Laos, and Viet Nam) where the virus had been detected; this cluster was independent of the lineage-A viruses (recovered from Sri Lanka, Malaysia, and Singapore) and the lineage-B viruses obtained in the Americas. Among 6 amino acid substitutions (i.e., 86R in protein C; 124L, 271T, and 489V in protein E; 142I in protein NS2A; and 864S in protein NS5) shared among isolates obtained in Thailand, Singapore, and Laos from 2013–2017, 86R in protein C was reported in Indian viruses isolated after 2004. The 86R residue is located on a conserved surface of protein C and constitutes a T-cell epitope [79]. Together, these findings may suggest that the spread of DENV-3 genotype III viruses to Southeast Asia originated from the outbreaks that occurred in India and Pakistan in 2003–2004 and 2005–2006, respectively [28, 29]. Our phylogeographical analysis at least partially supported this hypothesis (Fig 6B, S2 Fig and S4 Fig). However, other possibilities should be also considered and carefully evaluated in future research.

In conclusion, our data indicated that DENV in Thailand has been showing an increase in genotypic diversity. Apparent limitations of the present study include the nature of our sampling process, given that we obtained all of our specimens from a single hospital. In addition, we analyzed a relatively small numbers of specimens. Nevertheless, we were able to show that DENV-2 genotype Cosmopolitan has newly appeared as a genotype co-circulating with Asian-I, at least in the local area (adjacent to the capital city Bangkok) served by BIDI. It will be important to see whether DENV-2 genotype Cosmopolitan replaces the current local genotype (Asian-I) and whether Cosmopolitan might cause a new outbreak in Thailand, given that our data also showed that DENV-3 genotype II has been mostly replaced by DENV-3 genotype III. Further studies will be needed to see whether newly emerging DENV genotypes show augmented virulence in new regions, an effect that might facilitate new outbreaks.

## Supporting information

S1 Table Primers used in the present study. (PDF)

S2 Table Dengue virus envelope sequences used in the present study. (PDF)

S3 Table Dengue virus complete coding sequences used in the present study. (PDF)

S4 Table Model comparison using path and stepping-stone sampling. (PDF)

S1 Fig The migrations of temporal dynamic for DENV-2 genotype Cosmopolitan to Thailand. (PDF)

S2 Fig The migrations of temporal dynamics for DENV-3 genotype III to Thailand. (PDF)

S3 Fig Molecular clock analysis of DENV-2 genotype Cosmopolitan complete coding sequence. (PDF)

S4 Fig Molecular clock analysis of DENV-3 genotype III complete coding sequence. (PDF)
