# Supplementary material for: Emergence of genotype Cosmopolitan of dengue virus type 2 and genotype III of dengue virus type 3 in Thailand
Source: PLoS One. 2018 Nov 12;13(11):e0207220. doi: 10.1371/journal.pone.0207220 (PMC6231660; doi:10.1371/journal.pone.0207220)
Supplement: S1 Table — (PDF) [file pone.0207220.s001.pdf]

S1 Table Primers used in this study.

Fragment	RT primer	Sequence 5'-3'	PCR primer	Sequence 5'-3'
DENV1-5	d1a10[a]	TCTCTCYGGCTCRAAGAGGG	d1s1C[a]	*GATGAGGGAAGATGGGG*AGTTGTTAGTCTACGTGGAC
			d1a10[a]	TCTCTCYGGCTCRAAGAGGG
DENV1-3	d1a5B[a]	*TTTGTCGGTCTGGGGGGGTAT*AGAACCTGTTGATTCAACRGC	d1s12[a]	AAATGGCAGAGGCGCTCAAGGG
			d1a5B[a]	*TTTGTCGGTCTGGGGGGGTAT*AGAACCTGTTGATTCAACRGC
DENV2-5	DV2RT6817[b]	TGCGGCCACCACTGTGAGGATGGC	T7-5TDVFwClaI[b]	GGCATCGATTAATACGACTCACTATAGAGTTGTTAGTCTACGTGGAC CGACAAAG
			DV2RvNheI6731NotI[b]	GGCGCGGCCGCAGCAAAACTATGAGAAAAAACTCCA
DENV2-3	3T DV Rv[b]	AGAACCTGTTGATTCAACAGCACC	DV2FwClaI6608NheI[b]	GGCATCGATAGGGAAGATGACCCTGGGAATGTG
			3T-NotI DV Rv[b]	GCCGCGGCCGCAGAACCTGTTGATTCAACAGCACC
DENV3-5	d3a11[a]	TTGGTCCAGCCAGGATCA	d3s1C[a]	*GATGAGGGAAGATGGGG*AGTTGTTAGTCTACGTGGAC
			d3a11[a]	TTGGTCCAGCCAGGATCA
DENV3-3	d3a5B[a]	*TTTGTCGGTCTGGGGGGGTAT*AGAACCTGTTGATTCAACAGC	d3a5B[a]	*TTTGTCGGTCTGGGGGGGTAT*AGAACCTGTTGATTCAACAGC
			d3s12[a]	GCTCATGGAATTCAGGCAAT
DENV4-5	DV4-7148R[c]	ATTGCGTAATGGACTAAAAGCA	DV-1F[c]	AGTTGTTAGTCTACGTGGACCGACAA
			DV4-7130R[c]	AAGGTCGTTGGGTTCACTTG
DENV4-3	d4a5B[a]	*TTTGTCGGTCTGGGGGGGTAT*AGAACCTGTTGGATCAACAAC	DV4-6923F[c]	GGACGCTCTATGCAGTAGCC
			d4a5B[a]	*TTTGTCGGTCTGGGGGGGTAT*AGAACCTGTTGGATCAACAAC

Underlying represent restriction sites or T7 promoter sequences that are not present in dengue virus genomes.

[a] Christenbury, J. G., 2010

[b] Kurosu, T., 2010

[c] designed in the present study
