# Supplementary material for: Emergence of genotype Cosmopolitan of dengue virus type 2 and genotype III of dengue virus type 3 in Thailand
Source: PLoS One. 2018 Nov 12;13(11):e0207220. doi: 10.1371/journal.pone.0207220 (PMC6231660; doi:10.1371/journal.pone.0207220)
Supplement: S2 Table — (PDF) [file pone.0207220.s002.pdf]

**S2 Table. Dengue virus sequences used in present study.**

| Dataset | Serotype | Genotype | Accession number | Country | Year |
|---|---|---|---|---|---|
| Genotype classification | DENV-1 | I | AF350498 | China | 1980 |
| | DENV-1 | I | AB074760 | Japan | 1943 |
| | DENV-1 | I | AY732483 | Thailand | 1981 |
| | DENV-1 | I | AY732481 | Thailand | 1982 |
| | DENV-1 | I | AY732477 | Thailand | 1991 |
| | DENV-1 | I | AY732475 | Thailand | 1994 |
| | DENV-1 | I | AY732479 | Thailand | 2001 |
| | DENV-1 | I | AY732482 | Thailand | 2001 |
| | DENV-1 | I | AY732480 | Thailand | 2004 |
| | DENV-1 | I | HG316481 | Thailand | 2010 |
| | DENV-1 | I | KF887994 | Thailand | 2013 |
| | DENV-1 | I | KX595191 | Viet Nam | 2013 |
| | DENV-1 | II | AF180817 | Thailand | 1964 |
| | DENV-1 | III | EF457905 | Malaysia | 1972 |
| | DENV-1 | IV | KT827366 | China | 2007 |
| | DENV-1 | IV | DQ672560 | French Polynesia | 2001 |
| | DENV-1 | IV | AB189120 | Indonesia | 1998 |
| | DENV-1 | IV | KC762651 | Indonesia | 2007 |
| | DENV-1 | IV | GQ868602 | Philippines | 2004 |
| | DENV-1 | IV | DQ285560 | Reunion | 2004 |
| | DENV-1 | V | AF514889 | Argentina | 2000 |
| | DENV-1 | V | AF226685 | Brazil | 1990 |
| | DENV-1 | V | KF289072 | India | 2011 |
| | DENV-1 | V | AY722803 | Myanmar | 1998 |
| | DENV-1 | V | AY732474 | Thailand | 1980 |
| | DENV-1 | V | AY732476 | Thailand | 1980 |
| | DENV-1 | VI | KR919820 | Brunei | 2014 |
| | DENV-2 | American | AF119661 | China | 1985 |
| | DENV-2 | American | GQ868592 | Colombia | 1986 |
| | DENV-2 | American | HM582099 | Fiji | 1971 |
| | DENV-2 | American | HQ999999 | Guatemala | 2009 |
| | DENV-2 | American | KC294223 | Peru | 2010 |
| | DENV-2 | Asian-I | FJ639704 | Cambodia | 2003 |
| | DENV-2 | Asian-I | GU131932 | Cambodia | 2008 |
| | DENV-2 | Asian-I | NC001474 | Thailand | 1964 |
| | DENV-2 | Asian-I | GQ868542 | Thailand | 1994 |
| | DENV-2 | Asian-I | DQ181798 | Thailand | 1999 |
| | DENV-2 | Asian-I | FJ898452 | Thailand | 2003 |
| | DENV-2 | Asian-I | KY586563 | Thailand | 2006 |
| | DENV-2 | Asian-I | EU482784 | Viet Nam | 2003 |
| | DENV-2 | Asian-I | JF730049 | Viet Nam | 2007 |
| | DENV-2 | Asian-II | AF204177 | China | 1989 |
| | DENV-2 | Asian-II | AF204178 | China | 1987 |
| | DENV-2 | Asian-II | GQ398268 | Indonesia | 1975 |
| | DENV-2 | Asian-II | AF038403 | New Guinea | 1944 |
| | DENV-2 | Asian-II | HQ891024 | Taiwan | 2008 |
| | DENV-2 | Asian-II | JF730050 | USA | 2007 |
| | DENV-2 | Cosmopolitan | AY037116 | Australia | 1993 |
| | DENV-2 | Cosmopolitan | EU179857 | Brunei | 2005 |
| | DENV-2 | Cosmopolitan | EU056810 | Burkina Faso | 1983 |
| | DENV-2 | Cosmopolitan | AF359579 | China | 1999 |
| | DENV-2 | Cosmopolitan | AF276619 | China | 2000 |
| | DENV-2 | Cosmopolitan | FJ196853 | China | 2003 |
| | DENV-2 | Cosmopolitan | HM488257 | Guam | 2001 |
| | DENV-2 | Cosmopolitan | JQ955624 | India | 2011 |
| | DENV-2 | Cosmopolitan | JX475906 | India | 2009 |
| | DENV-2 | Cosmopolitan | GQ398258 | Indonesia | 1975 |
| | DENV-2 | Cosmopolitan | GQ398259 | Indonesia | 1976 |
| | DENV-2 | Cosmopolitan | GQ398260 | Indonesia | 1976 |
| | DENV-2 | Cosmopolitan | AB189122 | Indonesia | 1998 |
| | DENV-2 | Cosmopolitan | AY858035 | Indonesia | 2004 |
| | DENV-2 | Cosmopolitan | KJ010186 | Pakistan | 2013 |
| | DENV-2 | Cosmopolitan | EU081177 | Singapore | 2005 |
| | DENV-2 | Cosmopolitan | JF327392 | Singapore | 2009 |
| | DENV-2 | Cosmopolitan | FJ882602 | Sri Lanka | 1996 |
| | DENV-2 | Cosmopolitan | GQ252677 | Sri Lanka | 2004 |
| | DENV-2 | Cosmopolitan | DQ645546 | Taiwan | 2002 |
| | DENV-2 | Cosmopolitan | EU482672 | Viet Nam | 2006 |
| | DENV-3 | I | JN406515 | Australia | 2008 |
| | DENV-3 | I | JQ920480 | French Polynesia | 1996 |
| | DENV-3 | I | KC762686 | Indonesia | 2007 |
| | DENV-3 | I | JQ920486 | New Caledonia | 1996 |
| | DENV-3 | I | KU509279 | Philippines | 2008 |
| | DENV-3 | I | EU081223 | Singapore | 2005 |
| | DENV-3 | I | KX380839 | Singapore | 2012 |
| | DENV-3 | II | KC261634 | China | 2012 |
| | DENV-3 | II | GQ868593 | Thailand | 1973 |
| | DENV-3 | II | KU509280 | Thailand | 2011 |
| | DENV-3 | III | JX669490 | Brazil | 2002 |
| | DENV-3 | III | FJ882576 | Nicaragua | 1994 |
| | DENV-3 | III | KX380842 | Singapore | 2013 |
| | DENV-3 | III | GQ199887 | Sri Lanka | 1983 |
| | DENV-3 | III | NC_001475 | Sri Lanka | 2000 |
| | DENV-3 | III | DQ675531 | Taiwan | 1998 |
| | DENV-3 | III | FJ687448 | Thailand | 2001 |
| | DENV-3 | III | MF142763 | Thailand | 2015 |
| | DENV-3 | III | KF955460 | Viet Nam | 2008 |
| | DENV-3 | IV | L11433 | Puerto Rico | 1963 |
| | DENV-3 | IV | L11434 | Puerto Rico | 1977 |
| | DENV-3 | IV | L11439 | Tahiti | 1965 |
| | DENV-3 | V | EF629370 | Brazil | 2002 |
| | DENV-3 | V | JN697379 | Brazil | 2006 |
| | DENV-3 | V | AF317645 | China | 1980 |
| | DENV-3 | V | KM190937 | Philippines | 1964 |
| | DENV-3 | V | KU050695 | Philippines | 1956 |
| | DENV-3 | V | JQ922554 | USA | 1963 |
| | DENV-4 | I | JN638570 | Cambodia | 2008 |
| | DENV-4 | I | FJ196850 | China | 1990 |
| | DENV-4 | I | KU509287 | India | 2009 |
| | DENV-4 | I | KX845005 | India | 2015 |
| | DENV-4 | I | KF041260 | Pakistan | 2009 |
| | DENV-4 | I | KR011349 | Philippines | 1956 |
| | DENV-4 | I | KP792537 | Singapore | 2011 |
| | DENV-4 | I | AY618991 | Thailand | 1977 |
| | DENV-4 | I | AY618990 | Thailand | 1991 |
| | DENV-4 | I | AY618992 | Thailand | 2001 |
| | DENV-4 | I | KR922405 | Thailand | 2011 |
| | DENV-4 | IIA | JQ915083 | French Polynesia | 2007 |
| | DENV-4 | IIA | KC762694 | Indonesia | 2007 |
| | DENV-4 | IIA | KU523871 | Philippines | 2014 |
| | DENV-4 | IIA | AY618993 | Thailand | 2000 |
| | DENV-4 | IIB | KP188566 | Brazil | 2013 |
| | DENV-4 | IIB | GQ868585 | Colombia | 2005 |
| | DENV-4 | IIB | AF326573 | Dominican | 1981 |
| | DENV-4 | IIB | JF262782 | Haiti | 1994 |
| | DENV-4 | IIB | FJ850059 | Puerto Rico | 1998 |
| | DENV-4 | IIB | FJ639773 | Venezuela | 2001 |
| | DENV-4 | IIB | HQ332176 | Venezuela | 2007 |
| | DENV-4 | III | AY618989 | Thailand | 1997 |
| | DENV-4 | III | AY618988 | Thailand | 1997 |
| | DENV-4 | III | KY586945 | Thailand | 1998 |
| | DENV-4 | III | KY586946 | Thailand | 1998 |

| | | | | | |
|---|---|---|---|---|---|
| DENV-2 Thailand | DENV-2 | Asian-I | NC001474 | Thailand | 1964 |
| | DENV-2 | Asian-I | DQ181816 | Thailand | 1974 |
| | DENV-2 | Asian-I | GU289914 | Thailand | 1974 |
| | DENV-2 | Asian-I | DQ181814 | Thailand | 1976 |
| | DENV-2 | Asian-I | DQ181815 | Thailand | 1976 |
| | DENV-2 | Asian-I | DQ181812 | Thailand | 1977 |
| | DENV-2 | Asian-I | DQ181813 | Thailand | 1977 |
| | DENV-2 | Asian-I | DQ181810 | Thailand | 1978 |
| | DENV-2 | Asian-I | DQ181811 | Thailand | 1978 |
| | DENV-2 | Asian-I | DQ181805 | Thailand | 1979 |
| | DENV-2 | Asian-I | DQ181808 | Thailand | 1979 |
| | DENV-2 | Asian-I | DQ181826 | Thailand | 1980 |
| | DENV-2 | Asian-I | DQ181827 | Thailand | 1980 |
| | DENV-2 | Asian-I | DQ181828 | Thailand | 1980 |
| | DENV-2 | Asian-I | DQ181823 | Thailand | 1981 |
| | DENV-2 | Asian-I | DQ181824 | Thailand | 1981 |
| | DENV-2 | Asian-I | DQ181819 | Thailand | 1982 |
| | DENV-2 | Asian-I | DQ181822 | Thailand | 1982 |
| | DENV-2 | Asian-I | DQ181837 | Thailand | 1983 |
| | DENV-2 | Asian-I | DQ181838 | Thailand | 1983 |
| | DENV-2 | Asian-I | DQ181804 | Thailand | 1984 |
| | DENV-2 | Asian-I | DQ181834 | Thailand | 1984 |
| | DENV-2 | Asian-I | DQ181835 | Thailand | 1984 |
| | DENV-2 | Asian-I | DQ181803 | Thailand | 1985 |
| | DENV-2 | Asian-I | DQ181832 | Thailand | 1985 |
| | DENV-2 | Asian-I | DQ181833 | Thailand | 1985 |
| | DENV-2 | Asian-I | DQ181830 | Thailand | 1986 |
| | DENV-2 | Asian-I | DQ181831 | Thailand | 1986 |
| | DENV-2 | Asian-I | DQ181829 | Thailand | 1987 |
| | DENV-2 | Asian-I | DQ181848 | Thailand | 1987 |
| | DENV-2 | Asian-I | DQ181802 | Thailand | 1988 |
| | DENV-2 | Asian-I | DQ181847 | Thailand | 1988 |
| | DENV-2 | Asian-I | DQ181845 | Thailand | 1990 |
| | DENV-2 | Asian-I | DQ181887 | Thailand | 1990 |
| | DENV-2 | Asian-I | DQ181839 | Thailand | 1991 |
| | DENV-2 | Asian-I | DQ181840 | Thailand | 1991 |
| | DENV-2 | Asian-I | DQ181859 | Thailand | 1992 |
| | DENV-2 | Asian-I | DQ181855 | Thailand | 1993 |
| | DENV-2 | Asian-I | DQ181884 | Thailand | 1993 |
| | DENV-2 | Asian-I | DQ181851 | Thailand | 1994 |
| | DENV-2 | Asian-I | DQ181882 | Thailand | 1994 |
| | DENV-2 | Asian-I | GQ868542 | Thailand | 1994 |
| | DENV-2 | Asian-I | DQ181800 | Thailand | 1995 |
| | DENV-2 | Asian-I | DQ181849 | Thailand | 1995 |
| | DENV-2 | Asian-I | DQ181881 | Thailand | 1995 |
| | DENV-2 | Asian-I | DQ181866 | Thailand | 1996 |
| | DENV-2 | Asian-I | DQ181879 | Thailand | 1996 |
| | DENV-2 | Asian-I | GQ868545 | Thailand | 1996 |
| | DENV-2 | Asian-I | DQ181862 | Thailand | 1997 |
| | DENV-2 | Asian-I | DQ181899 | Thailand | 1997 |
| | DENV-2 | Asian-I | DQ181799 | Thailand | 1998 |
| | DENV-2 | Asian-I | DQ181860 | Thailand | 1998 |
| | DENV-2 | Asian-I | DQ181897 | Thailand | 1998 |
| | DENV-2 | Asian-I | DQ181798 | Thailand | 1999 |
| | DENV-2 | Asian-I | DQ181877 | Thailand | 1999 |
| | DENV-2 | Asian-I | DQ181895 | Thailand | 1999 |
| | DENV-2 | Asian-I | DQ181873 | Thailand | 2000 |
| | DENV-2 | Asian-I | DQ181894 | Thailand | 2000 |
| | DENV-2 | Asian-I | KY586654 | Thailand | 2000 |
| | DENV-2 | Asian-I | DQ181871 | Thailand | 2001 |
| | DENV-2 | Asian-I | EU117323 | Thailand | 2001 |
| | DENV-2 | Asian-I | EU117336 | Thailand | 2001 |
| | DENV-2 | Asian-I | EU117341 | Thailand | 2001 |
| | DENV-2 | Asian-I | EU117351 | Thailand | 2001 |
| | DENV-2 | Asian-I | KY586555 | Thailand | 2002 |
| | DENV-2 | Asian-I | KY586604 | Thailand | 2002 |
| | DENV-2 | Asian-I | KY586606 | Thailand | 2002 |
| | DENV-2 | Asian-I | KY586678 | Thailand | 2002 |
| | DENV-2 | Asian-I | FJ898452 | Thailand | 2003 |
| | DENV-2 | Asian-I | JF812102 | Thailand | 2003 |
| | DENV-2 | Asian-I | KY586547 | Thailand | 2003 |
| | DENV-2 | Asian-I | KY586562 | Thailand | 2003 |
| | DENV-2 | Asian-I | KY586615 | Thailand | 2003 |
| | DENV-2 | Asian-I | JQ993218 | Thailand | 2004 |
| | DENV-2 | Asian-I | JQ993223 | Thailand | 2004 |
| | DENV-2 | Asian-I | KY586598 | Thailand | 2004 |
| | DENV-2 | Asian-I | KY586605 | Thailand | 2004 |
| | DENV-2 | Asian-I | KY586612 | Thailand | 2004 |
| | DENV-2 | Asian-I | JQ993217 | Thailand | 2005 |
| | DENV-2 | Asian-I | JQ993226 | Thailand | 2005 |
| | DENV-2 | Asian-I | KY586566 | Thailand | 2005 |
| | DENV-2 | Asian-I | KY586590 | Thailand | 2005 |
| | DENV-2 | Asian-I | KY586563 | Thailand | 2006 |
| | DENV-2 | Asian-I | KY586565 | Thailand | 2006 |
| | DENV-2 | Asian-I | KY586575 | Thailand | 2006 |
| | DENV-2 | Asian-I | JN568273 | Thailand | 2007 |
| | DENV-2 | Asian-I | KY586596 | Thailand | 2007 |
| | DENV-2 | Asian-I | KY586587 | Thailand | 2008 |
| | DENV-2 | Asian-I | KY58662 | Thailand | 2008 |
| | DENV-2 | Asian-I | JF967990 | Thailand | 2009 |
| | DENV-2 | Asian-I | KY586597 | Thailand | 2009 |
| | DENV-2 | Asian-I | JF968033 | Thailand | 2010 |
| | DENV-2 | Asian-I | JF968045 | Thailand | 2010 |
| | DENV-2 | Asian-I | JN568245 | Thailand | 2010 |
| | DENV-2 | Asian-I | KY586625 | Thailand | 2010 |
| | DENV-2 | Asian-I | KF729020 | Thailand | 2011 |
| | DENV-2 | Asian-I | KF729021 | Thailand | 2011 |
| | DENV-2 | Asian-I | KF729022 | Thailand | 2011 |
| | DENV-2 | Asian-I | KU509273 | Thailand | 2011 |
| | DENV-2 | Asian-I | KY851468 | Thailand | 2012 |
| | DENV-2 | Asian-I | KY851478 | Thailand | 2012 |
| | DENV-2 | Asian-I | KY851496 | Thailand | 2012 |
| | DENV-2 | Asian-I | KT781555 | Thailand | 2013 |
| | DENV-2 | Asian-I | KY851466 | Thailand | 2013 |
| | DENV-2 | Asian-I | KY851469 | Thailand | 2013 |
| | DENV-2 | Asian-I | KY851502 | Thailand | 2013 |
| | DENV-2 | Asian-I | KY882539 | Thailand | 2013 |
| | DENV-2 | Asian-I | KT175135 | Thailand | 2014 |
| | DENV-2 | Asian-I | KX147335 | Thailand | 2014 |
| | DENV-2 | Cosmopolitan | AF410377 | Thailand | 1998 |
| | DENV-2 | Cosmopolitan | KT781537 | Thailand | 2012 |
| | DENV-2 | Cosmopolitan | KT781538 | Thailand | 2012 |
| | DENV-2 | Cosmopolitan | KJ545481 | Thailand | 2013 |
| | DENV-2 | Cosmopolitan | KT781572 | Thailand | 2014 |
| | DENV-2 | Cosmopolitan | KT806317 | Thailand | 2014 |
| | DENV-2 | Cosmopolitan | KY495805 | Thailand | 2016 |

| | | | | | |
|---|---|---|---|---|---|
| DENV-2 Asian-I | DENV-2 | Asian-I | NC001474 | Thailand | 1964 |
| | DENV-2 | Asian-I | GU289914 | Thailand | 1974 |
| | DENV-2 | Asian-I | DQ181805 | Thailand | 1979 |
| | DENV-2 | Asian-I | DQ181804 | Thailand | 1984 |
| | DENV-2 | Asian-I | DQ181803 | Thailand | 1985 |
| | DENV-2 | Asian-I | DQ181802 | Thailand | 1988 |
| | DENV-2 | Asian-I | GQ868542 | Thailand | 1994 |
| | DENV-2 | Asian-I | DQ181800 | Thailand | 1995 |
| | DENV-2 | Asian-I | GQ868545 | Thailand | 1996 |
| | DENV-2 | Asian-I | DQ181799 | Thailand | 1998 |
| | DENV-2 | Asian-I | DQ181798 | Thailand | 1999 |
| | DENV-2 | Asian-I | KY586654 | Thailand | 2000 |
| | DENV-2 | Asian-I | DQ181797 | Thailand | 2001 |
| | DENV-2 | Asian-I | KY586606 | Thailand | 2002 |
| | DENV-2 | Asian-I | FJ898452 | Thailand | 2003 |
| | DENV-2 | Asian-I | KY586605 | Thailand | 2004 |
| | DENV-2 | Asian-I | KY586563 | Thailand | 2006 |
| | DENV-2 | Asian-I | JN568273 | Thailand | 2007 |
| | DENV-2 | Asian-I | JF967990 | Thailand | 2009 |
| | DENV-2 | Asian-I | JF968045 | Thailand | 2010 |
| | DENV-2 | Asian-I | KU509273 | Thailand | 2011 |
| | DENV-2 | Asian-I | KY851467 | Thailand | 2012 |
| | DENV-2 | Asian-I | KY882539 | Thailand | 2013 |
| | DENV-2 | Asian-I | KJ806920 | Malaysia | 2013 |
| | DENV-2 | Asian-I | KJ806930 | Malaysia | 2014 |
| | DENV-2 | Asian-I | KX224266 | Singapore | 2014 |
| | DENV-2 | Asian-I | JN568261 | Australia | 2002 |
| | DENV-2 | Asian-I | JN568249 | Australia | 2003 |
| | DENV-2 | Asian-I | JN568246 | Australia | 2010 |
| | DENV-2 | Asian-I | KT781555 | Australia | 2013 |
| | DENV-2 | Asian-I | JQ815199 | China | 2009 |
| | DENV-2 | Asian-I | KF060920 | China | 2012 |
| | DENV-2 | Asian-I | MF459663 | China | 2013 |
| | DENV-2 | Asian-I | KP064517 | China | 2014 |
| | DENV-2 | Asian-I | KY849752 | Laos | 2008 |
| | DENV-2 | Asian-I | KY849757 | Laos | 2009 |
| | DENV-2 | Asian-I | KY849763 | Laos | 2010 |
| | DENV-2 | Asian-I | KY849768 | Laos | 2010 |
| | DENV-2 | Asian-I | LC147056 | Laos | 2013 |
| | DENV-2 | Asian-I | JF730044 | Cambodia | 2001 |
| | DENV-2 | Asian-I | KF921930 | Cambodia | 2002 |
| | DENV-2 | Asian-I | GQ868622 | Cambodia | 2003 |
| | DENV-2 | Asian-I | FJ639706 | Cambodia | 2004 |
| | DENV-2 | Asian-I | GQ868623 | Cambodia | 2005 |
| | DENV-2 | Asian-I | GU131927 | Cambodia | 2007 |
| | DENV-2 | Asian-I | GU131932 | Cambodia | 2008 |
| | DENV-2 | Asian-I | JF967986 | Cambodia | 2009 |
| | DENV-2 | Asian-I | KY495819 | Cambodia | 2015 |
| | DENV-2 | Asian-I | JF968026 | Myanmar | 2010 |
| | DENV-2 | Asian-I | KR051905 | Myanmar | 2013 |
| | DENV-2 | Asian-I | KT175136 | Myanmar | 2014 |
| | DENV-2 | Asian-I | KX357990 | Myanmar | 2015 |
| | DENV-2 | Asian-I | EU482782 | Viet Nam | 2003 |
| | DENV-2 | Asian-I | EU482774 | Viet Nam | 2004 |
| | DENV-2 | Asian-I | EU482777 | Viet Nam | 2005 |
| | DENV-2 | Asian-I | EU482465 | Viet Nam | 2006 |
| | DENV-2 | Asian-I | FJ205879 | Viet Nam | 2007 |
| | DENV-2 | Asian-I | FJ461309 | Viet Nam | 2008 |
| | DENV-2 | Asian-I | GU908495 | Viet Nam | 2009 |
| | DENV-2 | Asian-I | JN376793 | Viet Nam | 2010 |
| | DENV-2 | Asian-I | KY851484 | Viet Nam | 2011 |
| | DENV-2 | Asian-I | KY851491 | Viet Nam | 2012 |
| | DENV-2 | Asian-I | KT781567 | Viet Nam | 2014 |
| | DENV-2 | Asian-I | KY851494 | Viet Nam | 2013 |

| | | | | | |
|---|---|---|---|---|---|
| DENV-2 Cosmopolitan | DENV-2 | Cosmopolitan | JN568248 | Australia | 2003 |
| | DENV-2 | Cosmopolitan | JN568252 | Australia | 2008 |
| | DENV-2 | Cosmopolitan | JN568251 | Australia | 2006 |
| | DENV-2 | Cosmopolitan | JN568250 | Australia | 2004 |
| | DENV-2 | Cosmopolitan | JN568278 | Australia | 2003 |
| | DENV-2 | Cosmopolitan | KY495804.1 | Australia | 2016 |
| | DENV-2 | Cosmopolitan | JN036372 | Bangladesh | 2009 |
| | DENV-2 | Cosmopolitan | JN036379 | Bangladesh | 2005 |
| | DENV-2 | Cosmopolitan | JN036378 | Bangladesh | 2007 |
| | DENV-2 | Cosmopolitan | JN036377 | Bangladesh | 2008 |
| | DENV-2 | Cosmopolitan | JN036380 | Bangladesh | 2006 |
| | DENV-2 | Cosmopolitan | KT781532 | Bangladesh | 2011 |
| | DENV-2 | Cosmopolitan | EU056810 | Burkina Faso | 1983 |
| | DENV-2 | Cosmopolitan | KY627762 | Burkina Faso | 2016 |
| | DENV-2 | Cosmopolitan | KP191525 | China | 2014 |
| | DENV-2 | Cosmopolitan | KU550338 | China | 2015 |
| | DENV-2 | Cosmopolitan | KC964093 | China | 2001 |
| | DENV-2 | Cosmopolitan | KC964094 | China | 1993 |
| | DENV-2 | Cosmopolitan | JQ277886 | China | 2005 |
| | DENV-2 | Cosmopolitan | KT751353 | China | 2015 |
| | DENV-2 | Cosmopolitan | JX470186 | China | 2010 |
| | DENV-2 | Cosmopolitan | FJ158608 | China | 2007 |
| | DENV-2 | Cosmopolitan | KF060919 | China | 2012 |
| | DENV-2 | Cosmopolitan | KJ807797 | China | 2013 |
| | DENV-2 | Cosmopolitan | KX621247 | China | 2015 |
| | DENV-2 | Cosmopolitan | LC310791.1 | Cote d' Ivoire | 2017 |
| | DENV-2 | Cosmopolitan | JN568255 | East Timor | 2002 |
| | DENV-2 | Cosmopolitan | JN568254 | East Timor | 2000 |
| | DENV-2 | Cosmopolitan | KY275222 | East Timor | 2001 |
| | DENV-2 | Cosmopolitan | KY495815 | East Timor | 2003 |
| | DENV-2 | Cosmopolitan | JQ650022 | French Polynesia | 1996 |
| | DENV-2 | Cosmopolitan | JQ650026 | French Polynesia | 1997 |
| | DENV-2 | Cosmopolitan | JQ650027 | French Polynesia | 1998 |
| | DENV-2 | Cosmopolitan | KY782125 | French Polynesia | 2017 |
| | DENV-2 | Cosmopolitan | FJ538922 | India | 1983 |
| | DENV-2 | Cosmopolitan | FJ538923 | India | 1991 |
| | DENV-2 | Cosmopolitan | FJ538913 | India | 1997 |
| | DENV-2 | Cosmopolitan | FJ538911 | India | 1993 |
| | DENV-2 | Cosmopolitan | FJ538912 | India | 1995 |
| | DENV-2 | Cosmopolitan | FJ538924 | India | 1990 |
| | DENV-2 | Cosmopolitan | FJ538925 | India | 1992 |
| | DENV-2 | Cosmopolitan | KX061431 | India | 2015 |
| | DENV-2 | Cosmopolitan | DQ448236 | India | 2001 |
| | DENV-2 | Cosmopolitan | KJ545478 | India | 2013 |
| | DENV-2 | Cosmopolitan | JF967992 | India | 2009 |
| | DENV-2 | Cosmopolitan | JQ955624 | India | 2011 |
| | DENV-2 | Cosmopolitan | KU509271 | India | 2006 |
| | DENV-2 | Cosmopolitan | KY495802 | India | 2016 |
| | DENV-2 | Cosmopolitan | JN568260 | India | 2003 |
| | DENV-2 | Cosmopolitan | FJ538905 | India | 2004 |
| | DENV-2 | Cosmopolitan | FJ807633 | India | 2005 |
| | DENV-2 | Cosmopolitan | KY427084 | India | 2010 |
| | DENV-2 | Cosmopolitan | KT781565 | India | 2014 |
| | DENV-2 | Cosmopolitan | GQ398261 | Indonesia | 1976 |
| | DENV-2 | Cosmopolitan | GQ398260 | Indonesia | 1976 |
| | DENV-2 | Cosmopolitan | GQ398258 | Indonesia | 1975 |
| | DENV-2 | Cosmopolitan | GQ398259 | Indonesia | 1976 |
| | DENV-2 | Cosmopolitan | GQ398262 | Indonesia | 1976 |
| | DENV-2 | Cosmopolitan | LC027875 | Indonesia | 2012 |
| | DENV-2 | Cosmopolitan | JN568242 | Indonesia | 2009 |
| | DENV-2 | Cosmopolitan | KT012506 | Indonesia | 2011 |
| | DENV-2 | Cosmopolitan | GQ398263 | Indonesia | 1975 |
| | DENV-2 | Cosmopolitan | GQ398264 | Indonesia | 1976 |
| | DENV-2 | Cosmopolitan | KC762678 | Indonesia | 2010 |
| | DENV-2 | Cosmopolitan | JF967954 | Indonesia | 2008 |
| | DENV-2 | Cosmopolitan | KC762672 | Indonesia | 2008 |
| | DENV-2 | Cosmopolitan | KM216709 | Indonesia | 2011 |
| | DENV-2 | Cosmopolitan | KC762668 | Indonesia | 2008 |
| | DENV-2 | Cosmopolitan | KC762669 | Indonesia | 2007 |
| | DENV-2 | Cosmopolitan | KJ184316 | Indonesia | 2013 |
| | DENV-2 | Cosmopolitan | KT175123 | Indonesia | 2014 |
| | DENV-2 | Cosmopolitan | KU529751 | Indonesia | 2015 |
| | DENV-2 | Cosmopolitan | MG779194 | Kenya | 2017 |
| | DENV-2 | Cosmopolitan | KT716076 | Kenya | 2013 |
| | DENV-2 | Cosmopolitan | KJ806918 | Malaysia | 2013 |
| | DENV-2 | Cosmopolitan | JF968028 | Malaysia | 2010 |
| | DENV-2 | Cosmopolitan | KJ806776 | Malaysia | 2013 |
| | DENV-2 | Cosmopolitan | KX452026 | Malaysia | 2014 |
| | DENV-2 | Cosmopolitan | KJ806878 | Malaysia | 2012 |
| | DENV-2 | Cosmopolitan | KY851456 | Malaysia | 2011 |
| | DENV-2 | Cosmopolitan | KJ806773 | Malaysia | 2005 |
| | DENV-2 | Cosmopolitan | KJ806774 | Malaysia | 2007 |
| | DENV-2 | Cosmopolitan | JF967960 | Malaysia | 2008 |
| | DENV-2 | Cosmopolitan | JQ650032 | New Caledonia | 1997 |
| | DENV-2 | Cosmopolitan | JQ650036 | New Caledonia | 1998 |
| | DENV-2 | Cosmopolitan | JQ650038 | New Caledonia | 1999 |
| | DENV-2 | Cosmopolitan | KF041236 | Pakistan | 2008 |
| | DENV-2 | Cosmopolitan | KF041235 | Pakistan | 2009 |
| | DENV-2 | Cosmopolitan | KF360005 | Pakistan | 2010 |
| | DENV-2 | Cosmopolitan | KM217157 | Pakistan | 2011 |
| | DENV-2 | Cosmopolitan | KJ010186 | Pakistan | 2013 |
| | DENV-2 | Cosmopolitan | KY495810.1 | Papua New Guinea | 2016 |
| | DENV-2 | Cosmopolitan | KY794785 | Papua New Guinea | 2010 |
| | DENV-2 | Cosmopolitan | KU517845 | Papua New Guinea | 2013 |
| | DENV-2 | Cosmopolitan | JN568263 | Philippines | 2003 |
| | DENV-2 | Cosmopolitan | KF744397 | Philippines | 2001 |
| | DENV-2 | Cosmopolitan | KU509277 | Philippines | 2010 |
| | DENV-2 | Cosmopolitan | KU509269 | Philippines | 2009 |
| | DENV-2 | Cosmopolitan | KJ946239 | Philippines | 2008 |
| | DENV-2 | Cosmopolitan | KF744398 | Philippines | 2005 |
| | DENV-2 | Cosmopolitan | KT175126 | Philippines | 2014 |
| | DENV-2 | Cosmopolitan | KU517847 | Philippines | 2015 |
| | DENV-2 | Cosmopolitan | KY851414 | Philippines | 2012 |
| | DENV-2 | Cosmopolitan | KY851425 | Philippines | 2013 |
| | DENV-2 | Cosmopolitan | AB194884 | Philippines | 2004 |
| | DENV-2 | Cosmopolitan | LC416035.1 | Saudi Arabia | 2018 |
| | DENV-2 | Cosmopolitan | MF314189 | Singapore | 2016 |
| | DENV-2 | Cosmopolitan | GQ357716 | Singapore | 2007 |
| | DENV-2 | Cosmopolitan | GQ357761 | Singapore | 2008 |
| | DENV-2 | Cosmopolitan | JN380817 | Singapore | 2009 |
| | DENV-2 | Cosmopolitan | JN544362 | Singapore | 2011 |
| | DENV-2 | Cosmopolitan | JN030219 | Singapore | 2010 |
| | DENV-2 | Cosmopolitan | GQ357785 | Singapore | 2003 |
| | DENV-2 | Cosmopolitan | GQ357797 | Singapore | 2004 |
| | DENV-2 | Cosmopolitan | JN544399 | Singapore | 2011 |
| | DENV-2 | Cosmopolitan | KU948303 | Singapore | 2016 |
| | DENV-2 | Cosmopolitan | KX380818 | Singapore | 2012 |
| | DENV-2 | Cosmopolitan | KY495808.1 | Solomon | 2016 |
| | DENV-2 | Cosmopolitan | KC848581 | Somalia | 2011 |
| | DENV-2 | Cosmopolitan | FJ882602 | Sri Lanka | 1996 |
| | DENV-2 | Cosmopolitan | LC312197.1 | Sri Lanka | 2017 |
| | DENV-2 | Cosmopolitan | KY495803.1 | Sri Lanka | 2016 |
| | DENV-2 | Cosmopolitan | AB194883 | Sri Lanka | 2004 |
| | DENV-2 | Cosmopolitan | GQ252676 | Sri Lanka | 2003 |
| | DENV-2 | Cosmopolitan | EF016253 | Taiwan | 2002 |
| | DENV-2 | Cosmopolitan | JQ403524 | Taiwan | 2010 |
| | DENV-2 | Cosmopolitan | KT175112 | Taiwan | 2012 |
| | DENV-2 | Cosmopolitan | KU365903 | Taiwan | 2015 |
| | DENV-2 | Cosmopolitan | KT175114 | Taiwan | 2013 |
| | DENV-2 | Cosmopolitan | MG189962 | Tanzania | 2014 |
| | DENV-2 | Cosmopolitan | KT781566 | Thailand | 2014 |
| | DENV-2 | Cosmopolitan | KJ545481.1 | Thailand | 2013 |
| | DENV-2 | Cosmopolitan | KT781572 | Thailand | 2015 |
| | DENV-2 | Cosmopolitan | AF410377.1 | Thailand | 1998 |
| | DENV-2 | Cosmopolitan | KU509272 | Thailand | 2009 |
| | DENV-2 | Cosmopolitan | KT781537 | Thailand | 2012 |
| | DENV-2 | Cosmopolitan | KT781538.1 | Thailand | 2012 |
| | DENV-2 | Cosmopolitan | KY495805.1 | Thailand | 2016 |
| | DENV-2 | Cosmopolitan | KT806317 | Thailand | 2014 |
| | DENV-2 | Cosmopolitan | KY782126.1 | Vanuatu | 2017 |
| | DENV-2 | Cosmopolitan | KY971723 | Viet Nam | 2015 |
| | DENV-2 | Cosmopolitan | KT781567 | Viet Nam | 2014 |
| | DENV-2 | Cosmopolitan | EU482672 | Viet Nam | 2006 |

| | | | | | |
|---|---|---|---|---|---|
| DENV-3 Thailand | DENV-3 | II | AY676360 | Thailand | 1974 |
| | DENV-3 | II | AY676384 | Thailand | 1977 |
| | DENV-3 | II | AY676359 | Thailand | 1980 |
| | DENV-3 | II | AY676380 | Thailand | 1980 |
| | DENV-3 | II | AY676385 | Thailand | 1980 |
| | DENV-3 | II | AY676356 | Thailand | 1981 |
| | DENV-3 | II | AY676370 | Thailand | 1981 |
| | DENV-3 | II | AY676355 | Thailand | 1982 |
| | DENV-3 | II | KJ737430 | Thailand | 1983 |
| | DENV-3 | II | AY676358 | Thailand | 1983 |
| | DENV-3 | II | AY676372 | Thailand | 1983 |
| | DENV-3 | II | AY676371 | Thailand | 1984 |
| | DENV-3 | II | AY676401 | Thailand | 1984 |
| | DENV-3 | II | AY676368 | Thailand | 1985 |
| | DENV-3 | II | AY676415 | Thailand | 1985 |
| | DENV-3 | II | AY676418 | Thailand | 1985 |
| | DENV-3 | II | AY676354 | Thailand | 1986 |
| | DENV-3 | II | AY676374 | Thailand | 1986 |
| | DENV-3 | II | AY676352 | Thailand | 1987 |
| | DENV-3 | II | AY676353 | Thailand | 1987 |
| | DENV-3 | II | AY676362 | Thailand | 1987 |
| | DENV-3 | II | AY676378 | Thailand | 1987 |
| | DENV-3 | II | AY676379 | Thailand | 1987 |
| | DENV-3 | II | AY676417 | Thailand | 1988 |
| | DENV-3 | II | AY676419 | Thailand | 1988 |
| | DENV-3 | II | AY676361 | Thailand | 1990 |
| | DENV-3 | II | AY676421 | Thailand | 1990 |
| | DENV-3 | II | AY676367 | Thailand | 1991 |
| | DENV-3 | II | AY676416 | Thailand | 1991 |
| | DENV-3 | II | AY676386 | Thailand | 1992 |
| | DENV-3 | II | AY676350 | Thailand | 1993 |
| | DENV-3 | II | AY676351 | Thailand | 1993 |
| | DENV-3 | II | AY676357 | Thailand | 1993 |
| | DENV-3 | II | AY676375 | Thailand | 1993 |
| | DENV-3 | II | AY676381 | Thailand | 1993 |
| | DENV-3 | II | AY876494 | Thailand | 1994 |
| | DENV-3 | II | AY923865 | Thailand | 1994 |
| | DENV-3 | II | KJ737429 | Thailand | 1994 |
| | DENV-3 | II | AY676366 | Thailand | 1994 |
| | DENV-3 | II | AY676382 | Thailand | 1994 |
| | DENV-3 | II | AY676373 | Thailand | 1995 |
| | DENV-3 | II | AY676376 | Thailand | 1995 |
| | DENV-3 | II | AY676365 | Thailand | 1996 |
| | DENV-3 | II | AY676369 | Thailand | 1996 |
| | DENV-3 | II | AY676364 | Thailand | 1997 |
| | DENV-3 | II | AY676377 | Thailand | 1997 |
| | DENV-3 | II | AY676397 | Thailand | 1997 |
| | DENV-3 | II | AY676398 | Thailand | 1997 |
| | DENV-3 | II | AY676404 | Thailand | 1997 |
| | DENV-3 | II | AY676405 | Thailand | 1997 |
| | DENV-3 | II | AY676412 | Thailand | 1997 |
| | DENV-3 | II | DQ518661 | Thailand | 1997 |
| | DENV-3 | II | JN575574 | Thailand | 1997 |
| | DENV-3 | II | JN575575 | Thailand | 1997 |
| | DENV-3 | II | AY676348 | Thailand | 1998 |
| | DENV-3 | II | AY676349 | Thailand | 1998 |
| | DENV-3 | II | AY676388 | Thailand | 1998 |
| | DENV-3 | II | AY676389 | Thailand | 1998 |
| | DENV-3 | II | AY676391 | Thailand | 1998 |
| | DENV-3 | II | AY676392 | Thailand | 1998 |
| | DENV-3 | II | AY676393 | Thailand | 1998 |
| | DENV-3 | II | AY676395 | Thailand | 1998 |
| | DENV-3 | II | AY676396 | Thailand | 1998 |
| | DENV-3 | II | AY676399 | Thailand | 1998 |
| | DENV-3 | II | AY676400 | Thailand | 1998 |
| | DENV-3 | II | AY676403 | Thailand | 1998 |
| | DENV-3 | II | AY676406 | Thailand | 1998 |
| | DENV-3 | II | DQ518663 | Thailand | 1998 |
| | DENV-3 | II | AY676387 | Thailand | 1999 |
| | DENV-3 | II | AY676411 | Thailand | 1999 |
| | DENV-3 | II | AY676413 | Thailand | 1999 |
| | DENV-3 | II | AY676408 | Thailand | 2000 |
| | DENV-3 | II | AY676414 | Thailand | 2000 |
| | DENV-3 | II | FJ687448 | Thailand | 2001 |
| | DENV-3 | II | FJ744726 | Thailand | 2001 |
| | DENV-3 | II | FJ744727 | Thailand | 2001 |
| | DENV-3 | II | FJ744728 | Thailand | 2001 |
| | DENV-3 | II | FJ744729 | Thailand | 2001 |
| | DENV-3 | II | AY676394 | Thailand | 2001 |
| | DENV-3 | II | AY676402 | Thailand | 2001 |
| | DENV-3 | II | AY676407 | Thailand | 2001 |
| | DENV-3 | II | AY676409 | Thailand | 2001 |
| | DENV-3 | II | AY676410 | Thailand | 2001 |
| | DENV-3 | II | EU117353 | Thailand | 2001 |
| | DENV-3 | II | EU117354 | Thailand | 2001 |
| | DENV-3 | II | EU117356 | Thailand | 2001 |
| | DENV-3 | II | EU117358 | Thailand | 2001 |
| | DENV-3 | II | EU117364 | Thailand | 2001 |
| | DENV-3 | II | EU117369 | Thailand | 2001 |
| | DENV-3 | II | FJ744735 | Thailand | 2001 |
| | DENV-3 | II | FJ744740 | Thailand | 2001 |
| | DENV-3 | II | AY676383 | Thailand | 2002 |
| | DENV-3 | II | AY676420 | Thailand | 2002 |
| | DENV-3 | II | DQ518664 | Thailand | 2002 |
| | DENV-3 | II | DQ518660 | Thailand | 2003 |
| | DENV-3 | II | JQ993230 | Thailand | 2004 |
| | DENV-3 | II | JQ993228 | Thailand | 2005 |
| | DENV-3 | II | JF812103 | Thailand | 2006 |
| | DENV-3 | II | JF812104 | Thailand | 2006 |
| | DENV-3 | II | JF968084 | Thailand | 2009 |
| | DENV-3 | II | JF968093 | Thailand | 2010 |
| | DENV-3 | II | JF968094 | Thailand | 2010 |
| | DENV-3 | II | JN575576 | Thailand | 2010 |
| | DENV-3 | II | KT758740 | Thailand | 2011 |
| | DENV-3 | II | KT758741 | Thailand | 2011 |
| | DENV-3 | II | KT758742 | Thailand | 2011 |
| | DENV-3 | II | KT758748 | Thailand | 2012 |
| | DENV-3 | II | KT758790 | Thailand | 2013 |
| | DENV-3 | II | KT758791 | Thailand | 2013 |
| | DENV-3 | III | KY586820.1 | Thailand | 2008 |
| | DENV-3 | III | KY586822.1 | Thailand | 2009 |
| | DENV-3 | III | JF968092 | Thailand | 2010 |
| | DENV-3 | III | JF968098 | Thailand | 2010 |
| | DENV-3 | III | JF968106 | Thailand | 2010 |
| | DENV-3 | III | KY586821.1 | Thailand | 2010 |
| | DENV-3 | III | KT758771 | Thailand | 2013 |
| | DENV-3 | III | KU509302 | Thailand | 2013 |
| | DENV-3 | III | KY851612 | Thailand | 2013 |
| | DENV-3 | III | KY851614 | Thailand | 2013 |
| | DENV-3 | III | KY851616 | Thailand | 2013 |
| | DENV-3 | III | KY851617 | Thailand | 2013 |
| | DENV-3 | III | KY851618 | Thailand | 2013 |
| | DENV-3 | III | KY851619 | Thailand | 2013 |
| | DENV-3 | III | KY851621 | Thailand | 2013 |
| | DENV-3 | III | KU509303.1 | Thailand | 2013 |
| | DENV-3 | III | KP176708.1 | Thailand | 2013 |
| | DENV-3 | III | KY851615.1 | Thailand | 2013 |
| | DENV-3 | III | KY851613.1 | Thailand | 2013 |
| | DENV-3 | III | KY851611.1 | Thailand | 2013 |
| | DENV-3 | III | KU509304 | Thailand | 2014 |
| | DENV-3 | III | KT758784 | Thailand | 2015 |
| | DENV-3 | III | KY495823 | Thailand | 2015 |
| | DENV-3 | III | MF142763 | Thailand | 2015 |

| | | | | | |
|---|---|---|---|---|---|
| DENV-3 genotype III | DENV-3 | III | KC425219 | Brazil | 2002 |
| | DENV-3 | III | JX669504 | Brazil | 2006 |
| | DENV-3 | III | JX669499 | Brazil | 2004 |
| | DENV-3 | III | JX669493 | Brazil | 2005 |
| | DENV-3 | III | JX669490 | Brazil | 2002 |
| | DENV-3 | III | JF808124 | Brazil | 2003 |
| | DENV-3 | III | FJ898446 | Brazil | 2001 |
| | DENV-3 | III | EF629368 | Brazil | 1998 |
| | DENV-3 | III | EF629367 | Brazil | 1997 |
| | DENV-3 | III | EF629366 | Brazil | 1995 |
| | DENV-3 | III | AB111081 | Cambodia | 2000 |
| | DENV-3 | III | KF954945 | China | 2013 |
| | DENV-3 | III | JN662391 | China | 2009 |
| | DENV-3 | III | MH544651 | Colombia | 2016 |
| | DENV-3 | III | MH544647 | Colombia | 2015 |
| | DENV-3 | III | KY851579 | Colombia | 2012 |
| | DENV-3 | III | HM030558 | Colombia | 2009 |
| | DENV-3 | III | GU131954 | Colombia | 2006 |
| | DENV-3 | III | GU131950 | Colombia | 2001 |
| | DENV-3 | III | GQ868578 | Colombia | 2007 |
| | DENV-3 | III | GQ868577 | Colombia | 2005 |
| | DENV-3 | III | GQ868575 | Colombia | 2004 |
| | DENV-3 | III | FJ898443 | Colombia | 2003 |
| | DENV-3 | III | KT726346 | Cuba | 2002 |
| | DENV-3 | III | KT726340 | Cuba | 2001 |
| | DENV-3 | III | KT187295 | Djibouti | 2012 |
| | DENV-3 | III | KT187293 | Djibouti | 2011 |
| | DENV-3 | III | FJ898457 | Ecuador | 2000 |
| | DENV-3 | III | KU053472 | French Polynesia | 2013 |
| | DENV-3 | III | KU509281 | India | 2009 |
| | DENV-3 | III | KU216208 | India | 2013 |
| | DENV-3 | III | JQ922556 | India | 2005 |
| | DENV-3 | III | JQ686080 | India | 2010 |
| | DENV-3 | III | JQ686069 | India | 1966 |
| | DENV-3 | III | KT187283 | Cote d' Ivoire | 2008 |
| | DENV-3 | III | AB690858 | Japan | 2010 |
| | DENV-3 | III | MF370226 | Laos | 2013 |
| | DENV-3 | III | KF816163 | Laos | 2013 |
| | DENV-3 | III | LT898503 | Malaysia | 2011 |
| | DENV-3 | III | KP176709 | Malaysia | 2013 |
| | DENV-3 | III | JF968111 | Malaysia | 2010 |
| | DENV-3 | III | JF968068 | Malaysia | 2008 |
| | DENV-3 | III | FJ898440 | Mexico | 2003 |
| | DENV-3 | III | HM171540 | Mexico | 2006 |
| | DENV-3 | III | FJ898442 | Mexico | 2007 |
| | DENV-3 | III | FJ882575 | Mozambique | 1985 |
| | DENV-3 | III | KF973486 | Nicaragua | 2012 |
| | DENV-3 | III | GQ199864 | Nicaragua | 2008 |
| | DENV-3 | III | FJ882576 | Nicaragua | 1994 |
| | DENV-3 | III | KM217132 | Pakistan | 2013 |
| | DENV-3 | III | KF041259 | Pakistan | 2006 |
| | DENV-3 | III | KF041258 | Pakistan | 2009 |
| | DENV-3 | III | KF041255 | Pakistan | 2007 |
| | DENV-3 | III | KF041254 | Pakistan | 2008 |
| | DENV-3 | III | JF808129 | Paraguay | 2003 |
| | DENV-3 | III | JF808123 | Paraguay | 2002 |
| | DENV-3 | III | HQ235027 | Paraguay | 2007 |
| | DENV-3 | III | KJ189299 | Peru | 2005 |
| | DENV-3 | III | KJ189295 | Peru | 2006 |
| | DENV-3 | III | KJ189292 | Peru | 2009 |
| | DENV-3 | III | KJ189286 | Peru | 2008 |
| | DENV-3 | III | KJ189257 | Peru | 2004 |
| | DENV-3 | III | KJ189256 | Peru | 2002 |
| | DENV-3 | III | FJ547073 | Puerto Rico | 2000 |
| | DENV-3 | III | FJ547070 | Puerto Rico | 1998 |
| | DENV-3 | III | FJ547069 | Puerto Rico | 1999 |
| | DENV-3 | III | FJ182010 | Puerto Rico | 2005 |
| | DENV-3 | III | FJ024468 | Puerto Rico | 2004 |
| | DENV-3 | III | EU482564 | Puerto Rico | 2003 |
| | DENV-3 | III | KT187288 | Reunion | 2012 |
| | DENV-3 | III | KJ830751 | Saudi Arabia | 2014 |
| | DENV-3 | III | KU509282 | Senegal | 2009 |
| | DENV-3 | III | KY921907 | Singapore | 2015 |
| | DENV-3 | III | KX380842 | Singapore | 2013 |
| | DENV-3 | III | GU370053 | Singapore | 2007 |
| | DENV-3 | III | EU081182 | Singapore | 2005 |
| | DENV-3 | III | EU081181 | Singapore | 2004 |
| | DENV-3 | III | KC848589 | Somalia | 2011 |
| | DENV-3 | III | KX518579. | Sri Lanka | 2003 |
| | DENV-3 | III | KX518576 | Sri Lanka | 2004 |
| | DENV-3 | III | KU509283 | Sri Lanka | 2006 |
| | DENV-3 | III | KF955474 | Sri Lanka | 1989 |
| | DENV-3 | III | GQ252674 | Sri Lanka | 1997 |
| | DENV-3 | III | GQ199887 | Sri Lanka | 1983 |
| | DENV-3 | III | FJ882573 | Sri Lanka | 1993 |
| | DENV-3 | III | FJ189449 | Sri Lanka | 1990 |
| | DENV-3 | III | AY099336 | Sri Lanka | 2000 |
| | DENV-3 | III | KP176715 | Taiwan | 2012 |
| | DENV-3 | III | KP176714 | Taiwan | 2011 |
| | DENV-3 | III | DQ675533 | Taiwan | 1999 |
| | DENV-3 | III | AB549332 | Tanzania | 2010 |
| | DENV-3 | III | KY851612 | Thailand | 2013 |
| | DENV-3 | III | KY495823 | Thailand | 2015 |
| | DENV-3 | III | KU509304 | Thailand | 2014 |
| | DENV-3 | III | KU509302 | Thailand | 2013 |
| | DENV-3 | III | KT758784 | Thailand | 2015 |
| | DENV-3 | III | KT758771 | Thailand | 2013 |
| | DENV-3 | III | KP176707 | Thailand | 2012 |
| | DENV-3 | III | JF968106 | Thailand | 2010 |
| | DENV-3 | III | GQ868586 | Venezuela | 2007 |
| | DENV-3 | III | FJ639800 | Venezuela | 2004 |
| | DENV-3 | III | KP176711 | Viet Nam | 2013 |
