# Supplementary material for: Emergence of genotype Cosmopolitan of dengue virus type 2 and genotype III of dengue virus type 3 in Thailand
Source: PLoS One. 2018 Nov 12;13(11):e0207220. doi: 10.1371/journal.pone.0207220 (PMC6231660; doi:10.1371/journal.pone.0207220)
Supplement: S3 Table — (PDF) [file pone.0207220.s003.pdf]

S3 Table. Dengue virus complete coding sequences used in the present study.

Dataset	Serotype	Genotype	Lineage	Accession Number	Country	Year
DENV-2 Cosmopolitan	DENV-2	Cosmopolitan	C	KX372564	Australia	2015
	DENV-2	Cosmopolitan	A	EU056810	Burkina Faso	1983
	DENV-2	Cosmopolitan	A	GU131843	Burkina Faso	1986
	DENV-2	Cosmopolitan	A	KY627762	Burkina Faso	2016
	DENV-2	Cosmopolitan	B	AF276619	China	2000
	DENV-2	Cosmopolitan	B	AF359579	China	1999
	DENV-2	Cosmopolitan	C	JX470186	China	2010
	DENV-2	Cosmopolitan	C	KC131142	China	2012
	DENV-2	Cosmopolitan	C	KP012546	China	2014
	DENV-2	Cosmopolitan	C	KX621247	China	2015
	DENV-2	Cosmopolitan	C	HM488257	Guam	2001
	DENV-2	Cosmopolitan	B	DQ448231	India	2001
	DENV-2	Cosmopolitan	B	FJ898454	India	2006
	DENV-2	Cosmopolitan	B	JQ955623	India	2009
	DENV-2	Cosmopolitan	B	JQ955624	India	2011
	DENV-2	Cosmopolitan	C	AB189122	Indonesia	1998
	DENV-2	Cosmopolitan	A	GQ398258	Indonesia	1975
	DENV-2	Cosmopolitan	A	GQ398259	Indonesia	1976
	DENV-2	Cosmopolitan	A	GQ398260	Indonesia	1976
	DENV-2	Cosmopolitan	C	AY858035	Indonesia	2004
	DENV-2	Cosmopolitan	C	KC762676	Indonesia	2007
	DENV-2	Cosmopolitan	C	KC762670	Indonesia	2007
	DENV-2	Cosmopolitan	C	KC762672	Indonesia	2008
	DENV-2	Cosmopolitan	C	KU509268	Indonesia	2009
	DENV-2	Cosmopolitan	C	KC762680	Indonesia	2010
	DENV-2	Cosmopolitan	C	KU517846	Indonesia	2014
	DENV-2	Cosmopolitan	C	KX452026	Malaysia	2014
	DENV-2	Cosmopolitan	B	KF041236	Pakistan	2008
	DENV-2	Cosmopolitan	B	KF360005	Pakistan	2010
	DENV-2	Cosmopolitan	B	KJ010186	Pakistan	2013
	DENV-2	Cosmopolitan	C	KU517845	Papua New Guinea	2013
	DENV-2	Cosmopolitan	C	KU509269	Philippines	2009
	DENV-2	Cosmopolitan	C	KU509277	Philippines	2010
	DENV-2	Cosmopolitan	C	KU517847	Philippines	2015
	DENV-2	Cosmopolitan	B	KJ830750	Saudi Arabia	2014
	DENV-2	Cosmopolitan	C	JN851131	Singapore	2005
	DENV-2	Cosmopolitan	C	KM279580	Singapore	2013
	DENV-2	Cosmopolitan	C	KM279604	Singapore	2008
	DENV-2	Cosmopolitan	C	KU948303	Singapore	2016
	DENV-2	Cosmopolitan	C	KY921904	Singapore	2014
	DENV-2	Cosmopolitan	B	FJ882602	Sri Lanka	1996
	DENV-2	Cosmopolitan	B	GQ252676	Sri Lanka	2003
	DENV-2	Cosmopolitan	B	GQ252677	Sri Lanka	2004
	DENV-2	Cosmopolitan	C	DQ645546	Taiwan	2002
	DENV-2	Cosmopolitan	C	KU365902	Taiwan	2015
	DENV-2	Cosmopolitan	C	KU509272	Thailand	2009
	DENV-2	Cosmopolitan	C	EU482672	Vietnam	2006
DENV-3 genotype III	DENV-3	III	B	JX669508	Brazil	2006
	DENV-3	III	B	JF808120	Brazil	2009
	DENV-3	III	B	JX669490	Brazil	2002
	DENV-3	III	C	KF954945	China	2013
	DENV-3	III	C	JN662391	China	2009
	DENV-3	III	B	GQ868577	Colombia	2005
	DENV-3	III	B	KT726346	Cuba	2002
	DENV-3	III	B	KT726340	Cuba	2001
	DENV-3	III	C	KU216208	India	2013
	DENV-3	III	C	GQ466079	India	2008
	DENV-3	III	C	KU509281	India	2009
	DENV-3	III	C	FJ644564	India	2007
	DENV-3	III	C	KU509286	India	2011
	DENV-3	III	C	JQ922556	India	2005
	DENV-3	III	C	MF370226	Laos	2013
	DENV-3	III	B	AY099337	Martinique	1999
	DENV-3	III	B	FJ898442	Mexico	2007
	DENV-3	III	B	KF973486	Nicaragua	2012
	DENV-3	III	B	GQ199864	Nicaragua	2008
	DENV-3	III	B	FJ882576	Nicaragua	1994
	DENV-3	III	C	KF041258	Pakistan	2009
	DENV-3	III	C	KF041254	Pakistan	2008
	DENV-3	III	C	KF041255	Pakistan	2007
	DENV-3	III	C	KF041259	Pakistan	2006
	DENV-3	III	B	JF808129	Paraguay	2003
	DENV-3	III	B	KJ189286	Peru	2008
	DENV-3	III	B	KJ189265	Peru	2004
	DENV-3	III	B	EU482564	Puerto Rico	2003
	DENV-3	III	B	EU596494	Puerto Rico	2007
	DENV-3	III	A	KJ830751	Saudi Arabia	2014
	DENV-3	III	C	KU509282	Senegal	2009
	DENV-3	III	C	KY921907	Singapore	2015
	DENV-3	III	C	KX380842	Singapore	2013
	DENV-3	III	A	EU081181	Singapore	2004
	DENV-3	III	A	GU370053	Singapore	2007
	DENV-3	III	A	EU081182	Singapore	2005
	DENV-3	III	A	KF955474	Sri Lanka	1989
	DENV-3	III	A	GQ252674	Sri Lanka	1997
	DENV-3	III	A	GQ199887	Sri Lanka	1983
	DENV-3	III	A	FJ882573	Sri Lanka	1993
	DENV-3	III	A	AY099336	Sri Lanka	2000
	DENV-3	III	A	KU509283	Sri Lanka	2006
	DENV-3	III	A	DQ675533	Taiwan	1999
	DENV-3	III	C	MF142763	Thailand	2015
	DENV-3	III	B	GQ868586	Venezuela	2007
	DENV-3	III	B	FJ639800	Venezuela	2004
