# Supplementary material for: Emergence of genotype Cosmopolitan of dengue virus type 2 and genotype III of dengue virus type 3 in Thailand
Source: PLoS One. 2018 Nov 12;13(11):e0207220. doi: 10.1371/journal.pone.0207220 (PMC6231660; doi:10.1371/journal.pone.0207220)
Supplement: S4 Table — (PDF) [file pone.0207220.s004.pdf]

S4 Table. Model comparison using path and stepping-stone sampling.

Data set	Model combinations	Path sampling		Stepping-stone sampling	
		Log Marginal likelihood	Ranking	Log Marginal likelihood	Ranking
DENV-2 Asian-I Envelope Sequence	UCLN, Constant	-6283.15	4	-6283.36	4
	UCLN, Exponential	-6281.1	3	-6281.63	2
	UCLN, Bayesian Skyline	-6276.8	1	-6277	1
	UCLN, GMRF	-6281.08	2	-6281.82	3
DENV-2 Cosmopolitan Envelope Sequence	UCLN, Constant	-10518.06	4	-10520.29	4
	UCLN, Exponential	-10504.84	3	-10508.58	3
	UCLN, Bayesian Skyline	-10496.8	2	-10501.42	2
	UCLN, GMRF	-10476.76	1	-10478.4	1
DENV-3 Genotype III Envelope Sequence	UCLN, Constant	-6947.39	3	-6947.78	3
	UCLN, Exponential	-6942.25	2	-6942.27	2
	UCLN, Bayesian Skyline	-6936.4	1	-6938.03	1
	UCLN, GMRF	-6951.28	4	-6952.62	4
