# Supplementary material for: Emergence of genotype Cosmopolitan of dengue virus type 2 and genotype III of dengue virus type 3 in Thailand
Source: PLoS One. 2018 Nov 12;13(11):e0207220. doi: 10.1371/journal.pone.0207220 (PMC6231660; doi:10.1371/journal.pone.0207220)
Supplement: S1 Fig — (PDF) [file pone.0207220.s005.pdf]

S1 Fig

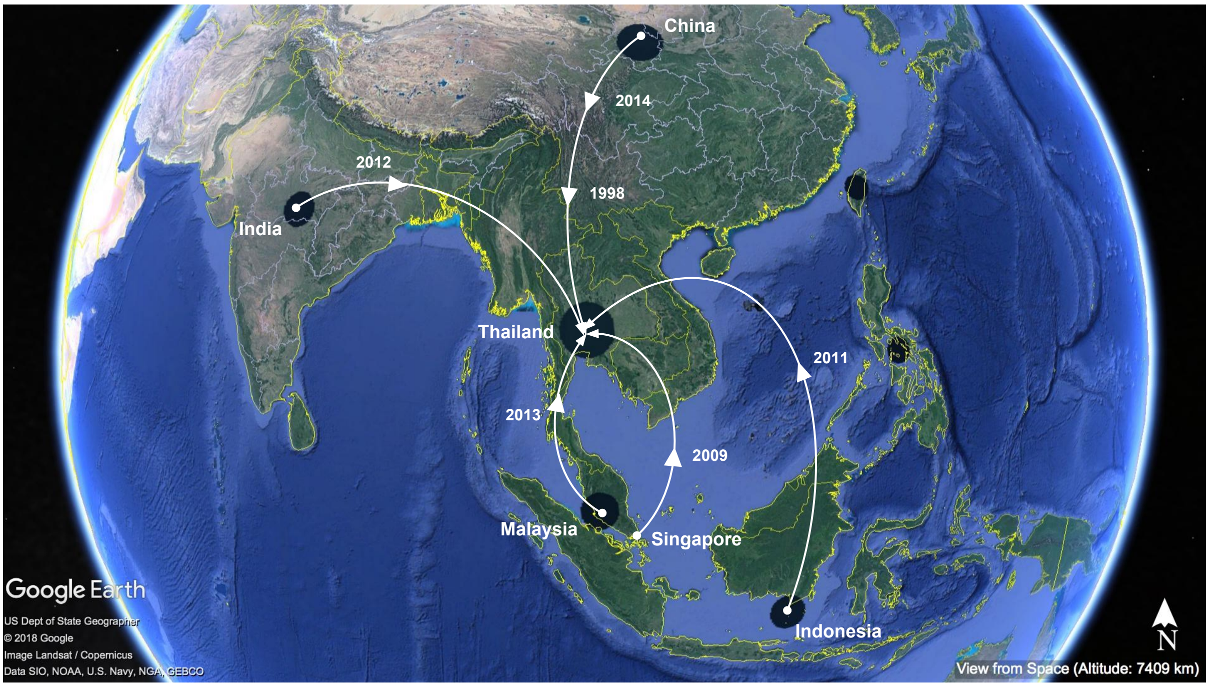

S1 Fig. The migrations of temporal dynamic for DENV-2 genotype Cosmopolitan to Thailand. The visualizing location annotated Maximum Clade Credibility tree reconstruction by SPREAD software. The branches express an overview of the possible routes of DENV-2 Cosmopolitan introduction into Thailand. The spread time and direction of the virus from China, India, Singapore, Malaysia, and Indonesia to Thailand is indicated with arrows. Map data: Google Earth Pro.
