# Supplementary material for: Emergence of genotype Cosmopolitan of dengue virus type 2 and genotype III of dengue virus type 3 in Thailand
Source: PLoS One. 2018 Nov 12;13(11):e0207220. doi: 10.1371/journal.pone.0207220 (PMC6231660; doi:10.1371/journal.pone.0207220)
Supplement: S2 Fig — (PDF) [file pone.0207220.s006.pdf]

S2 Fig

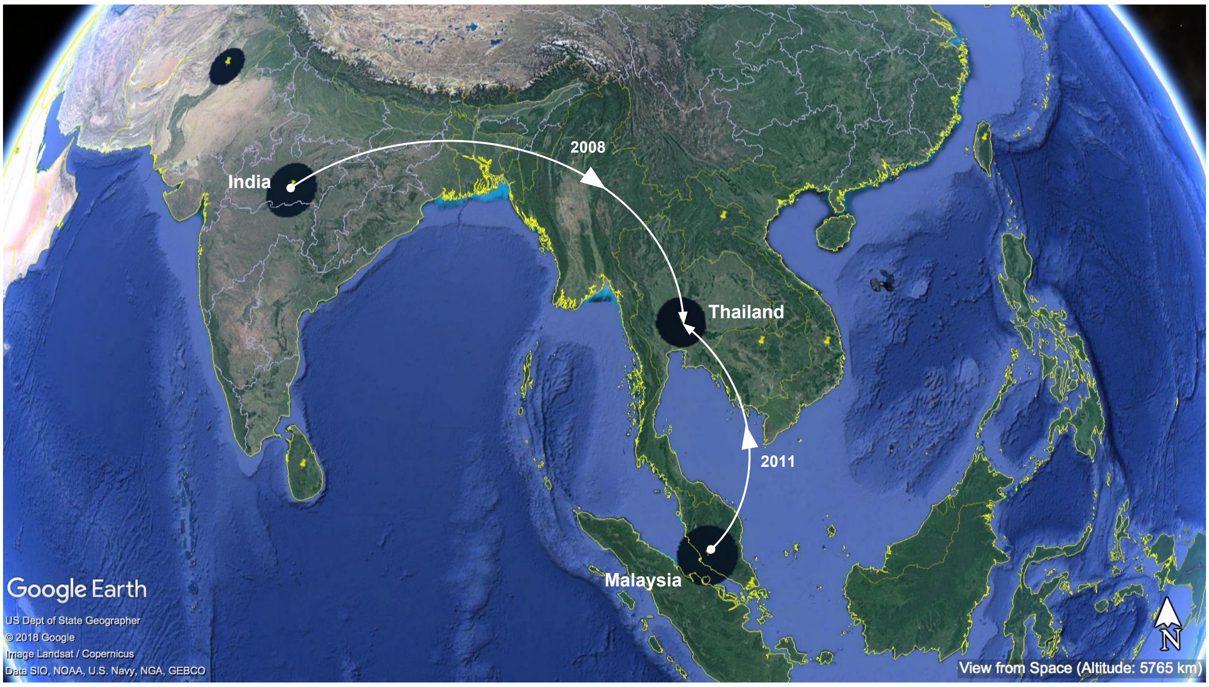

S2 Fig. The migrations of temporal dynamics for DENV-3 genotype III to Thailand. The visualizing location annotated Maximum Clade Credibility tree reconstruction by SPREAD software. The branches express an overview of the possible routes of DENV-3 genotype III introduction into Thailand. The spread time and direction of virus from India and Malaysia is indicated with arrows. Map data: Google Earth Pro.
