# Supplementary material for: Emergence of genotype Cosmopolitan of dengue virus type 2 and genotype III of dengue virus type 3 in Thailand
Source: PLoS One. 2018 Nov 12;13(11):e0207220. doi: 10.1371/journal.pone.0207220 (PMC6231660; doi:10.1371/journal.pone.0207220)
Supplement: S3 Fig — (PDF) [file pone.0207220.s007.pdf]

S3 Fig

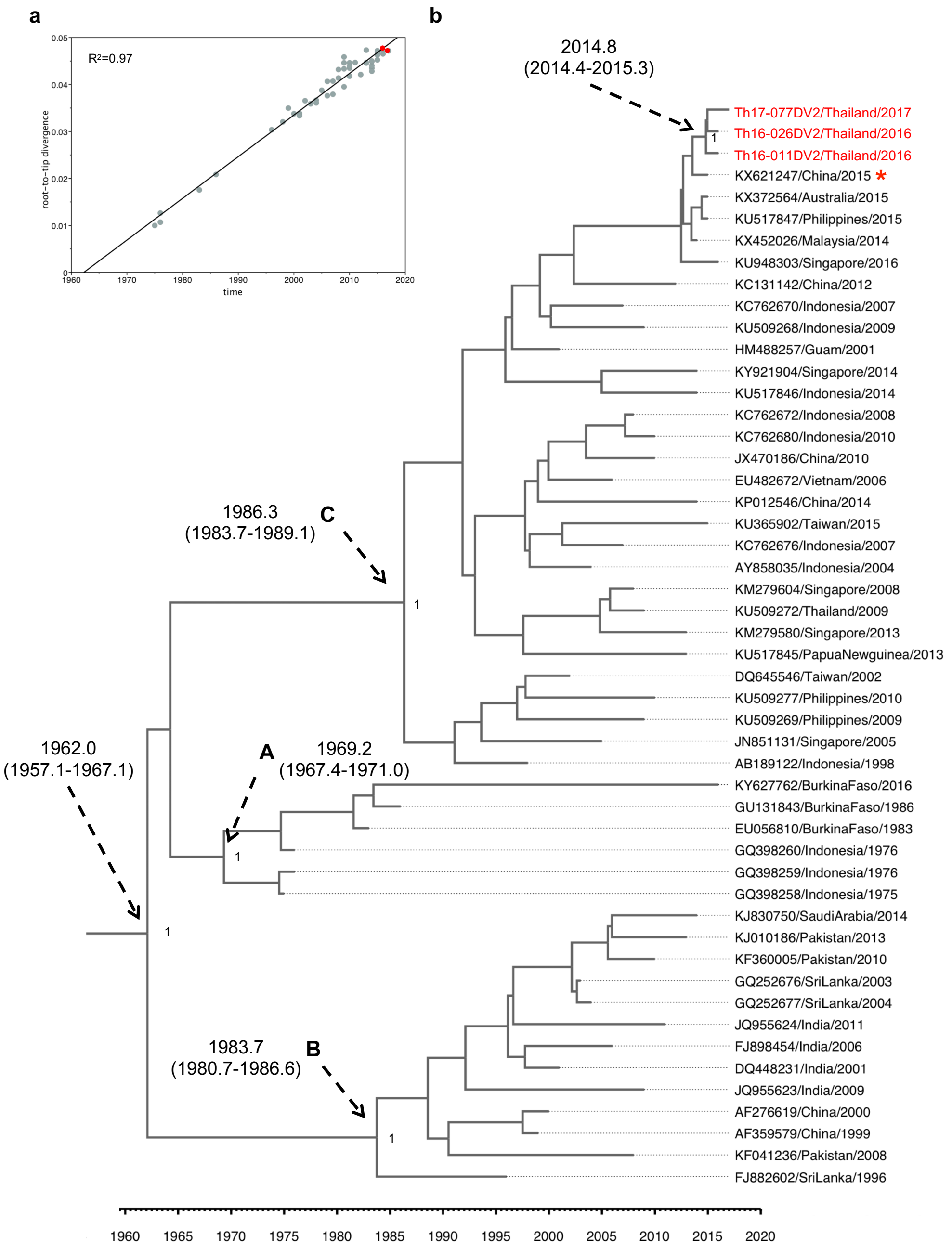

S3 Fig. Molecular clock analysis of DENV-2 genotype Cosmopolitan complete coding sequence. The dataset of DENV-2 genotype Cosmopolitan was composed of 3 sequences obtained in the present study and 47 sequences retrieved from GenBank (S3 Table). **(a)** Correlation of collection year and divergence from maximum likelihood tree. R^2, (coefficient of determination) = 0.97 was estimated using TempEst (shown at top left). Sequences obtained in the present study are shown as red dots. **(b)** Bayesian maximum clade credibility (MCC) phylogenetic tree estimated using BEAST v1.8.4. The mean time of the most recent common ancestor (tMRCA) and 95% highest probability density (HPD) are indicated with black dashed arrows shown in years, and posterior probability values are indicated adjacent to the node of interest. The name of each taxon is presented as accession number, country, and year of collection. Sequences obtained in the present study are labeled in red. Lineages A, B, and C are shown to the right. The KX621247 virus is indicated by a red asterisk.
